# Global transcriptional analysis suggests *Lasiodiplodia theobromae* pathogenicity factors involved in modulation of grapevine defensive response

**DOI:** 10.1186/s12864-016-2952-3

**Published:** 2016-08-11

**Authors:** Marcos Paolinelli-Alfonso, José Manuel Villalobos-Escobedo, Philippe Rolshausen, Alfredo Herrera-Estrella, Clara Galindo-Sánchez, José Fabricio López-Hernández, Rufina Hernandez-Martinez

**Affiliations:** 1 Departamento de Microbiología, Centro de Investigación Científica y de Educación Superior de Ensenada (CICESE), Ensenada, BC 22860 Mexico; 2 Laboratorio Nacional de Genómica para la Biodiversidad (LANGEBIO), Centro de Investigación y de Estudios Avanzados del I. P. N., Irapuato, Gto 36821 Mexico; 3 Department of Botany and Plant Sciences,University of California Riverside, Riverside, 92521 CA USA; 4 Departamento de Biotecnología Marina, Centro de Investigación Científica y de Educación Superior de Ensenada (CICESE), Ensenada, BC 22860 Mexico

**Keywords:** Fungal gene expression, Botryosphaeria dieback, *Vitis vinifera*, RT-qPCR, Graevine vascular diseases, RNA-seq, Transcriptome, Melanin

## Abstract

**Background:**

*Lasiodiplodia theobromae* is a fungus of the Botryosphaeriaceae that causes grapevine vascular disease, especially in regions with hot climates. Fungi in this group often remain latent within their host and become virulent under abiotic stress. Transcriptional regulation analysis of *L. theobromae* exposed to heat stress (HS) was first carried out in vitro in the presence of grapevine wood (GW) to identify potential pathogenicity genes that were later evaluated for *in planta* expression.

**Results:**

A total of 19,860 *de novo* assembled transcripts were obtained, forty-nine per cent of which showed homology to the Botryosphaeriaceae fungi, *Neofusicoccum parvum* or *Macrophomina phaseolina*. Three hundred ninety-nine have homology with genes involved in pathogenic processes and several belonged to expanded gene families in others fungal grapevine vascular pathogens. Gene expression analysis showed changes in fungal metabolism of phenolic compounds; where genes encoding for enzymes, with the ability to degrade salicylic acid (SA) and plant phenylpropanoid precursors, were up-regulated during in vitro HS response, in the presence of GW. These results suggest that the fungal L-tyrosine catabolism pathway could help the fungus to remove phenylpropanoid precursors thereby evading the host defense response. The *in planta* up-regulation of salicylate hydroxylase, intradiol ring cleavage dioxygenase and fumarylacetoacetase encoding genes, further supported this hypothesis. Those genes were even more up-regulated in HS-stressed plants, suggesting that fungus takes advantage of the increased phenylpropanoid precursors produced under stress. Pectate lyase was up-regulated while a putative amylase was down-regulated *in planta*, this could be associated with an intercellular growth strategy during the first stages of colonization.

**Conclusions:**

*L. theobromae* transcriptome was established and validated. Its usefulness was demonstrated through the identification of genes expressed during the infection process. Our results support the hypothesis that heat stress facilitates fungal colonization, because of the fungus ability to use the phenylpropanoid precursors and SA, both compounds known to control host defense.

**Electronic supplementary material:**

The online version of this article (doi:10.1186/s12864-016-2952-3) contains supplementary material, which is available to authorized users.

## Background

In recent years, global climate change has had a devastating impact on crop productivity. Drought and heat stress have resulted in an increased tree mortality worldwide [1–3]. This phenomenon could be attributed in part to endophytic fungi. Fungi inhabiting the vascular system of trees can become pathogenic under abiotic stress. In susceptible plant hosts, this results in the development of wood necrosis and cankers, because trees failed to effectively compartmentalize the pathogen. As the fungus colonizes its host, the plant vascular function becomes increasingly impaired. Prolonged abiotic stress conditions and biotic infections lead to tree decline and eventually death [4–6]. Two independent studies have analyzed, at the molecular level, the interaction of trees susceptible to fungal pathogens. Both studies highlighted the adaptation capability of the fungus to metabolize terpenoids and stilbene, the main defensive compounds produced by the host in response to infection, and use them as carbon sources for wood colonization [7, 8]. Both compounds are produced in response to biotic and abiotic stress in plants [9, 10], suggesting that the fungal colonization is favored when plants are under abiotic stress.

Grapes are the world’s most economically important fruit crop and have been cultivated in a broad range of environmental conditions [11]. Fungal vascular diseases (a.k.a. Eutypa dieback, Botryosphaeria dieback and Esca) are major factors limiting grape productivity and fruit marketability worldwide [12, 13]. Several taxonomically unrelated fungi are known to cause these diseases. *Lasiodiplodia theobromae* [teleomorph *Botryosphaeria rhodina* (Griff. & Maubl., Bull. Soc.Mycol. Fr. 25: 57. 1909)] is one of the causal agents of Botryosphaeria dieback. It is especially predominant in hot climates, and it has been classified as one of the most aggressive vascular pathogen of grapevine [12, 14–16]. Despite its socio-economic impact, there is still little knowledge on its biology and no genome sequence information is currently available. In comparison, *Eutypa lata* (Pers.:Fr.) Tul. & C. Tul. (syn. *E. armeniacae* Hansf. and Carter)*,* which causes similar wood symptoms, has been extensively studied and its genome has been sequenced [17–21]. *E. lata* has the capacity to colonize the host vascular system targeting non-structural carbohydrate (i.e. starch) and structural hemicellulosic cell wall glucans for its metabolism [20].

*L. theobromae* and related members of the family Botryosphaeriaceae have been recognized as latent pathogens in many hosts [22, 23]. This pathogen was identified as a causal agent of mortality in dogwood tree under drought stress [24]. Similarly, Álvarez-Loayza et al. [25] observed that the disease incidence caused by Botryosphaeriaceae, *Diplodia mutila* on tropical palm *Iriarte deltoidea,* had a clear correlation with light availability, higher plant exposure to sunlight results in faster disease progression. Authors suggest that fungal reactive oxygen species (ROS) detoxification and melanin production triggered the imbalance in the endophyte-host interaction.

Biochemical assays have been used traditionally to identify pathogenicity factors produced by grapevine vascular pathogens, including secondary metabolites and plant cell wall degrading enzymes (PCWDEs) [17, 18, 26]. The advent of high throughput (OMICs) technologies have allowed for a better understanding of the complex plant/pathogen interactions in grapevine diseases. For instance, the secretome analysis of *Diplodia seriata* identified three necrotic inducible proteins, suggesting that fungi induce a hypersensitive-like response in host grapevine cells [27]. Recently, the genomes of grapevine vascular pathogens *Neofusicoccum parvum*, *D. seriata* and *E. lata* were obtained [21, 28, 29]. Through a genomic comparative study, the expansion of dioxygenase (PF00775), pectate lyase (PF03211), major facilitator superfamily (PF07690), carboxylesterase (PF00135) and glucose-methanol-choline oxidoreductase (C-terminal: PF05199 and N-terminal:PF00732) gene families were identified on these pathogens suggesting a role in pathogenicity [29].

The hot and arid viticulture production areas, provide a unique place to study how commensal interaction between an endophytic fungus and grapevine, become pathogenic under extreme heat conditions. This research aim was to evaluate *L. theobromae* global transcriptional response in both in in vitro and *in planta* bioassays. In addition, this work was designed to better understand the impact of heat stress at the fungal transcriptional level, and how it affects pathogenicity and the expression of disease symptom in grapevines.

## Methods

### Fungal growth condition

*L. theobromae* UCD256Ma was isolated from grapevine showing symptoms of Botryosphaeria dieback located in Madera County in California, USA by Urbez-Torres et al. [14] and kindly shared by Dr. Douglas Gubler. The isolate was maintained at—20 °C in 20 % glycerol in the laboratory of phytopathology at CICESE. Infection and recovery in *Vitis vinifera* cv. Cabernet Sauvignon green shoots was used to reactive it. After recovery, a plug of mycelium of the fungus grown on Potato Dextrose Agar (PDA, Difco) was inoculated in Erlenmeyer flasks containing Vogel’s salts (VS) [30] or Vogel’s salts with chips of grapevine wood (GW). Wood samples were obtained from branches of one year old *V. vinifera* cv. Cabernet Sauvignon grown in greenhouse from cuttings, pieces were frozen in liquid nitrogen, ground with a blender (Waring), filtered through a 0.35 mm sieve (Precision Scientific) and autoclaved. After inoculation, flasks were incubated at 28 °C in darkness, without agitation for 48 h. Then, some flasks were exposed to HS by transferring them at 42 °C for 1 h and then returned to 28 °C, while controls were maintained at 28 °C. Ten minutes after HS, mycelia were collected using previously sterilized tweezers, washed with water treated with diethylpyrocarbonate (DEPC, Sigma) and transferred to 1.5 ml tubes with 500 μl of Nucleic Acid Preservation (NAP) buffer [31] and stored at −80 °C until RNA extraction.

### RNA extraction and sequencing

**Fig. 1:**
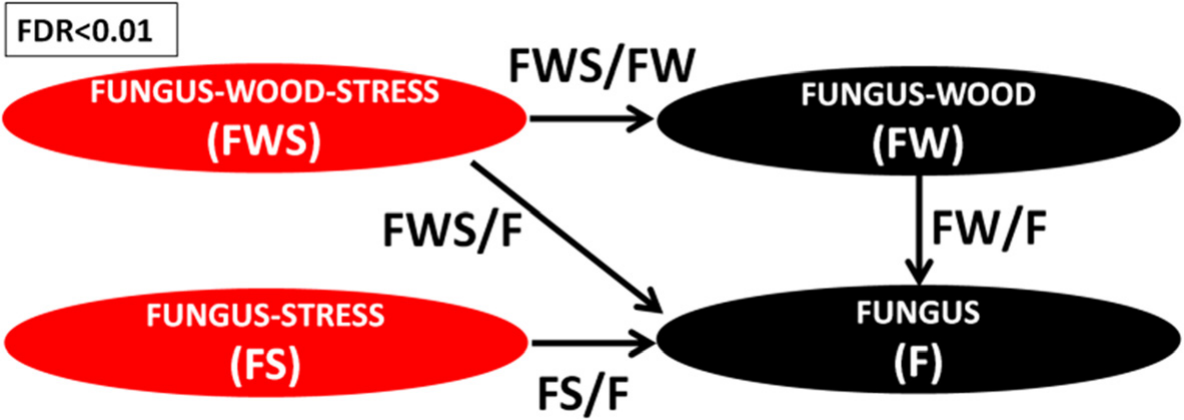
Contrasting conditions in RNAseq evaluated in general linear model statistics to identify differentially expressed genes. The conditions evaluated were from fungus grown in Vogel’s salts without heat stress (F) or under heat stress (FS), Vogel’s salts with 1 % of grapevine wood (GW), without heat stress (FW) or under heat stress (FWS). To evaluate the gene expression under the effect of heat stress (HS), the expression of FS was compared to F (FS/F). To test only the effect of HS when GW was present, FWS/FW ratio was measured, while to test both effects at the same time the ratio FWS/F was considered. Finally, fungal gene expression only as effect of GW, was measured evaluating FW/F ratio

Total RNA extraction was done through modification of protocols based on Reid et al. [32] and Vasanthaiah et al. [33] as described by Paolinelli-Alfonso et al. [34]. RQ1 DNAse (Promega) treatment was done according to manufacturer’s instructions. DNA-free total RNA was sent to the National Laboratory of Genomics for Biodiversity service facilities (Irapuato, Mexico) for sequencing. RNA quality was verified on an Agilent 2100 Bioanalyzer using the RNA 6000 Nano kit according to the manufacturer’s instructions. Complementary DNA (cDNA) and the ligation of adapters were done with the TruSeq Paired-End Cluster Kit v3-cBot-HS (Illumina) according to the manufacturer’s instruction. Libraries of cDNA were made from fungus grown in the conditions described in Fig. 1. These conditions were: grown in the absence of GW (F), in the absence of GW plus HS (FS), in the presence of GW (FW) and in the presence of GW plus HS (FWS). Three biological replicates for each condition were sequenced in 1 lane of an Illumina Hiseq2500, to obtain 100 bp pair-end reads. All sequence data are available from the Gene Expression Omnibus DataSets (GSE75978).

### Bioinformatics analysis

The general pipeline used for transcriptome analysis is schematically represented in Additional file 1: Figure S1. All scripts employed here are available upon request.

#### *De novo* transcriptome assembly and functional annotation

All the reads obtained from the Illumina sequencing were filtered using Trimmomatic (Trimmomatic-0.32, [35]) and sequencing quality control program (FastQC [36]) to select only those showing a PHRED quality higher than Q30, absence of adapters and homopolymers, and those with a length higher than 90 bp. The filtered sequences were used for assembly using Trinity (trinityrnaseq_r20140717, [37]) with the default settings (kmer = 25). The assembly quality check was done with the Perl script (analyze_blastPlus_topHit_coverage.pl) included in the trinityrnaseq_r20140717 package [38]. The fasta file output produced by Trinity, containing all possible isoforms, was filtered to retain only the longest using an online available Python script (longest_isoform_filter_trinity_assem.py). The longest isoform sequences were used as queries in BlastX (version 2.2.29+, [39]) from the National Center for Biotechnology Information (NCBI) using the non-redundant (NR) protein database, retaining the highest scored hit with E value < 0.001. The file with BlastX hits was used to retrieve the functional annotations with Blast2GO software (version 3.0, GO-DB version 2014–09, [38]) using the gene ontology (GO) database updated on September 2014 and default parameters as recommended [40]. The InterProScan sequence analysis application [41] in the Blast2GO software was used to predict the secretion signal with SignalP 4.0 [42]. The presence of transmembrane domains was determined with TMHMM [43]. Domain information was retrieved through PfamA [44] and HAMAP [45]. Sequences with positive SignalP predictions [41] in the N-terminal region and without any transmembrane regions as predicted by TMHMM [43] were selected as candidate secreted proteins.

The fasta file of the longest isoform sequences was used to predict the open reading frames (ORFs) through the usage of TransDecoder (TransDecoder_r20140704; [46]). The predicted ORFs sequences were used in a local BlastP (version 2.2.28+, [38]) analysis in the Pathogen-Host Interaction (PHI) database (version 3.6, [47]).

#### Differential expression and functional enrichment analysis

The raw reads of both ends obtained for each output file were aligned to the longest isoform transcripts using Bowtie 2 (version 2.1.0, [48]) with default parameters. The resulting output in SAM format was converted to BAM format through Samtools (version 0.1.19-96b5f2294a; [49]). Using the sorted bam file, the number of alignments were counted for each transcript and sample using a homemade Perl script specially designed (count_filebam.pl and count_names.pl) to output a tab delimited table. The statistical analysis was done using edgeR package version 3.4.2 [50] of Bioconductor [51] in the R environment (version 3.0.2, [52]). Normalization was done using counts of reads per million (cpm) and filtered to retain transcripts that had at least 3 cpm in more than 2 samples. The statistical analysis was done using a General Linear Model (GLM) approach. The conditions compared are shown in Fig. 1. The false discovery rate (FDR) < 0.01 was set as the threshold to consider genes as differentially expressed.

Differentially expressed genes (DEGs) for any of the contrasting conditions evaluated were arranged in clusters based on their expression pattern through Pearson correlations in edgeR. The list of genes for each cluster was used as test and all the differentially expressed genes were used as references in an enrichment analysis (Fisher’s exact test, FDR < 0.05) using Blast2GO software (version 3.0, GO-DB version 2014–09, [39]). The GOplot package (version 1.0.1, [53]) in the R environment was used to generate the graph GOcluster and GOcircle in order to obtain a more detailed view of co-regulated genes in relationship with functional enrichment.

### Evaluation of *L. theobromae* growth in benzoate

To evaluate the capacity of *L. theobromae* to grow in benzoic acid (BZA) as the sole carbon source, the fungus was inoculated in agar plates containing Vogel’s Minimal Medium (VMM) [30] supplemented with 0, 15 or 30 mM of BZA. Photographs were taken after 8 days of incubation at 28 °C.

### In vitro and *in planta* fungal gene expression of selected targets

Twelve genes that were found to be differentially expressed in the RNAseq analysis were selected for validation and relative quantification of gene expression through reverse transcription-quantitative PCR (RT-qPCR), both in vitro and *in planta* (genes selected and primers are shown in Additional file 2: Table S1).

The effect of HS was simulated in growth chambers by exposing the *L. theobromae*-infected plants to a daily temperature cycles changing from higher minimal to a higher maximal. One year-old cuttings *V. vinifera* cv. Merlot, were grown in UC special mix soil in 5 L pots and kept at room temperature at the University of California Riverside lathhouse facilities. Using a sterile razor blade, green shoots from thirty plants were wounded on the second basal internode and inoculated with a 3 mm mycelia plug obtained from a three day-old culture of *L. theobromae*. A total of 6 plants were inoculated with a PDA plug and used as controls. All wounds were covered with paraffin wax film (Parafilm M) following inoculation. Five days later, fifteen plants inoculated with *L. theobromae* and three plants inoculated with PDA were exposed to HS using night-day cycles ranging from 20 °C to 42 °C at 50 % humidity on growth chamber (Environmental Growth Chamber EGC-TC2). The remaining plants were incubated in a growth chamber (Conviron PGR15) with night-day cycles ranging from 10 °C to 30 °C at 50 % humidity, and used as controls without HS. At 7, 11 and 15 days post inoculation (dpi) plants were collected, shoots were cut in transversal sections at least 5 mm up or down from the inoculation point, a section was used for stereo microscopy (Leica M125) observation and the remaining were collected in NAP solution and stored at −20 °C until RNA extraction. The RNA extraction was done using a cetyltrimethylammonium bromide (CTAB) -based protocol (details in Additional file 3). Total RNA was treated with RQI DNAse (Promega) and the cDNA obtained with the ImProm AM3800 (Promega) kit, according to the manufacturer’s instruction. The qPCR was done on a CFX96 thermocycler (Bio-Rad) using 3 μl of cDNA and 7 μl of mastermix containing 2.5 mM MgCl2, 1X Taq polymerase Accustart buffer (Quanta), 0.2 mM of dNTPs, 0.03 U/ul Taq polymerase Accustart (Quanta), 1X EVAgreen (Biotium) and 0.2 μM of each primer. Two biological with three technical replicates were used for in vitro and three biological with three technical replicates were used for *in planta* gene expression analyses.

The HTqPCR package (version 3.2, [54]) for R environment (version 3.0.2, [52]) was used for normalization against β-tubulin mRNA, relative expression quantification and limma statistical analysis. *In planta* fungal gene expression was calculated as fold change in HS-treated host or non HS-treated and infected plants compared to *L. theobromae* growing in in vitro conditions. cDNA from non-infected plants was used as negative controls when the specificity was verified through the analysis of melting curves for each gene.

## Results

### Transcriptome assembly and functional annotations

Sequencing reads varied in number, ranging from 3 to 6 million per sample, with an average of approximately 5 million reads (Additional file 4: Figure S2), showing PHRED quality scores per base of 36. The *de novo* assembly with Trinity [37] produced 29,621 total components (including isoforms), %GC = 56 and N50 = 3,135 bp (details in Table 1).

**Table 1:** Main characteristics of Trinity *de novo* transcriptome assembly

Parameters	All transcript	Only longest Isoform
Number of contigs	29621	19860
Contig N10	6773	5525
Contig N20	5345	4321
Contig N30	4365	3572
Contig N40	3667	2971
Contig N50	3135	2472
Median contig length	1242	658
Average contig	1798.4	1310.58
Total assembled bases	53270493	27680863

From the 29,621 total components, the longest isoforms were selected, rendering 19,860 unique transcripts with N50 = 2,472 bp; the median length was 658 bp and the average contig length was 1,310.58 bp (Table 1). More than 50 % of the assembled transcripts showed at least 80 % full-length coverage as compared to well-annotated proteins, and 33 % showed 100 % coverage, indicating that the assembly fulfills the requirements of quality analysis through ortholog hit ratio [55] which justifies its use to map the reads and analyze gene expression.

When the transcripts were arranged according to the taxonomic information retrieved from BlastX, most hits showed similarity with members of either of the Botryosphaeriaceae family, *M. phaseolina* (5,800 sequences, 29 %) and *N. parvum* (3,800 sequences, 19 %), the only two Botryosphaeriaceae genomes available in the NR-NCBI database at the time of analysis, or several other fungi from the Ascomycota phylum (Additional file 5: Figure S3). The high number of *L. theobromae* sequences homologous to *M. phaseolina* over *N. parvum* was supported by previous phylogenetic analyses showing *L. theobromae* phylogenetically closer to *M. phaseolina* than to *N. parvum* [56].

From the 19,860 longest isoforms, 7,547 sequences (38 %) had recognizable functional GO annotation (details of annotation results in Additional file 6: Figure S4). The GO terms associated with each transcript encoded protein were used to associate them according to their function. A wide diversity of functions were identified (details in Additional file 7). Among others, in the Biological process in fourth level of gene ontology classification, most of the transcripts were involved in nitrogen metabolism (684), aromatic (665), organic cycles (689) and heterocycle (676) compounds metabolism. Also there are 589 transcripts involved in oxidation-reduction process.

Based on the importance of genes encoding proteins with extracellular functions in the establishment of the interaction of the fungus with its host, transcripts encoding proteins with N-terminal secretion signal were identified. From 15,981 transcripts that contain at least one ORF, 850 were predicted to be secreted.

**Fig. 2:**
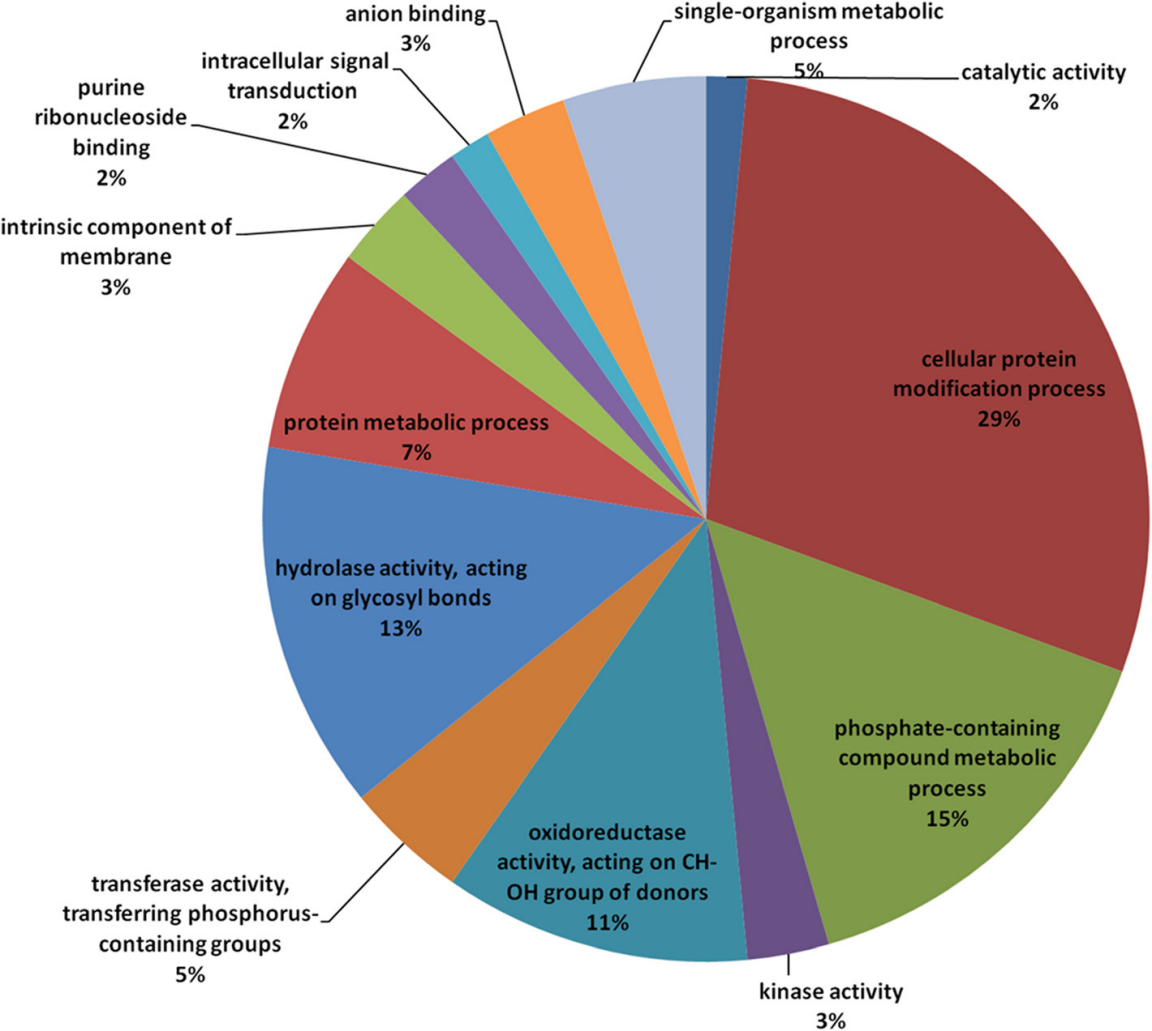
Pie chart describing the main Gene Ontology (GO) of molecular functions categories of transcripts with putative pathogenicity role. The predicted open reading frames encoded by transcripts were used in a local Blastp to find hits in the experimentally verified Pathogen-Host Interaction database (PHI-base). The PHI-base hits were classified based on its GO information

On other hand, a total of 399 protein-coding transcripts, showing homology with those included in the Pathogen-Host Interaction database (PHI-base) were identified. PHI-base contains experimentally verified pathogenicity or virulence factors, therefore *L. theobromae* transcripts showing PHI-base hits are predicted to be involved in pathogenicity. Based on functional annotation of PHI-base hits, they were classified according to their gene ontology information, and were identified as being involved in protein modification processes, metabolism of phosphate-containing compounds, hydrolase activity on glycosyl bonds, and oxidoreductase on CHOH groups (Fig. 2). Additionally, 22 PHI-base hits were predicted to be secreted, and according to their annotation, they could be involved in carbohydrate metabolism, proteolysis and oxido-reduction processes (Additional file 8).

### Differential gene expression, co-regulation and functional enrichment

**Fig. 3:**
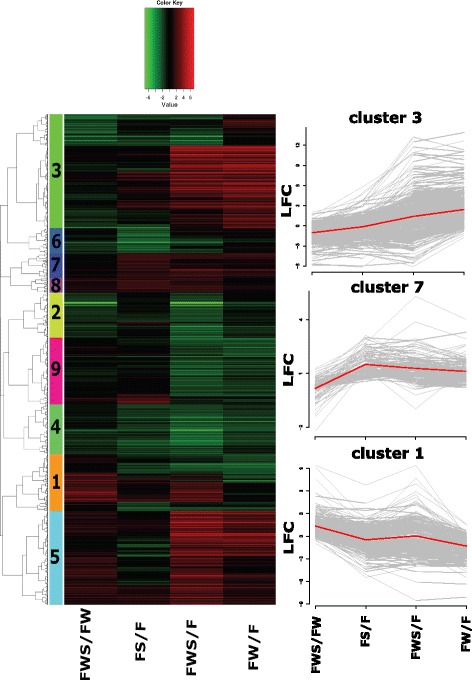
Heatmap showing clusters of co-regulated genes with their expression profiles. The dendrogram indicates the relationship between gene expression profiles as determined by hierarchical clustering (Pearson correlation). The differentially colored boxes at the left indicate different clusters of genes with similar expression profiles. The color key indicates logFC values ranging from bright red for most up-regulated to bright green for the most down-regulated genes, considering a FDR < 0.01. The expression pattern for clusters with functional enrichment (clusters 1, 3 and 7) are shown in a graph with gray lines for each gene while the average for all the genes in the cluster is shown in red line. The contrasting conditions evaluated are those indicated in Fig. 1. Briefly, FS/F: effect of heat stress (HS); FWS/FW: effect of HS but in the presence of grapevine wood; FWS/F: effect of HS in the presence of grapevine wood and FW/F: effect of only GW

The analysis of RNAseq data indicated that sufficient differences were achieved among treatments and therefore allowed the identification of DEGs (Additional file 9). In total, 2,386 DEGs (FDR < 0.01) were identified in any of the contrasting conditions tested (Fig. 1). These corresponded to 12 % of the total number of transcripts. Different numbers of DEGs were obtained for the different contrasting conditions, FWS/F contrast has the highest number (Table 2 and Additional file 10). All the contrast generate specific unique DEGs not shared with the DEGs from the other contrasts (Additional file 11: Figure S5). An analysis of their pattern of expression was used to arrange the genes in clusters of co-regulated genes (Fig. 3 and Additional file 12).

**Table 2:** Number of differentially expressed genes (DEGs, FDR < 0.01) and enrichment test for secretion for each contrasting conditions

Contrasting conditions	Regulation	Differentially expressed genes (DEGs)	Predicted secreted DEGs	FDR (hypergeometric test for enrichment of secreted proteins)
FWS/F	Up-regulated	905	83	0.156003
	Down-regulated	887	74	0.549571
FWS/FW	Up-regulated	173	13	0.707640
	Down-regulated	233	17	0.770976
FW/F	Up-regulated	565	61	0.012635
	Down-regulated	424	47	0.554427
FS/F	Up-regulated	203	23	0.077195
	Down-regulated	366	28	0.738934

Functional enrichment was obtained in three clusters of co-regulated genes. An enrichment for oxido-reductase activity was obtained for cluster 1 (FDR = 1.2x10^−3^), and the shared pattern of expression for the putative genes was mainly the induction in FWS/FW and FWS/F, and repression in FW/F (Fig. 3). Cluster 3 showed enrichment in hydrolase activity, mainly involved in the hydrolysis of O-glycosyl compounds (FDR = 2.3x10^−8^), and showed an inverse pattern of expression compared to cluster 1, with major protein-coding genes up-regulated in FW/F, but down-regulated in FWS/FW and a lower level of induction or down-regulation in FS/F (Fig. 3). Cluster 7 showed an enrichment (FDR < 0.01) in translation processes, GTPase and methyltransferase activities. The shared pattern of expression in cluster 7 was mainly due to the induction in FS/F (Fig. 3).

**Fig. 4:**
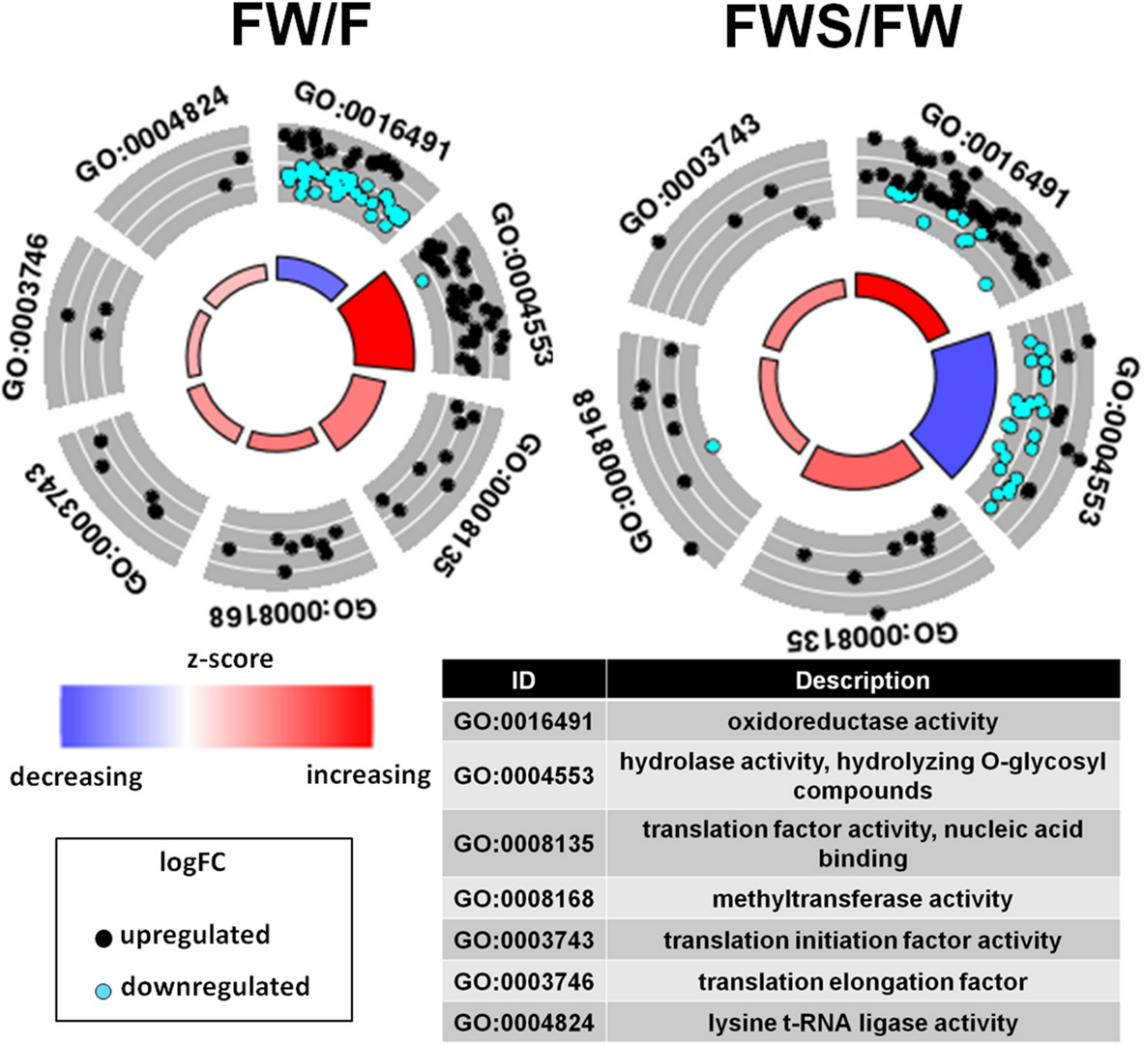
Gene Ontology (GO) of molecular functions categories with enrichment in hierarchical clusters of similar expression profiles. The GO term differential expression is considered through z-score ($\mathrm{zscore}=\frac{\left(\mathrm{upregulated}\hbox{-} \mathrm{downregulated}\right)}{\sqrt{\left(\mathrm{upregulated}+\mathrm{downregulated}\right)}}$, described in [53]). Red color indicates higher proportion of up-regulated genes and blue color corresponds to higher proportion of down-regulated genes. The color key indicates logFC values ranging from golden for up-regulated genes, to cyan for down-regulated genes. The effects of only grapevine wood (FW/F) and heat stress in the presence of grapevine wood (FWS/FW) were evaluated

**Fig. 5:**
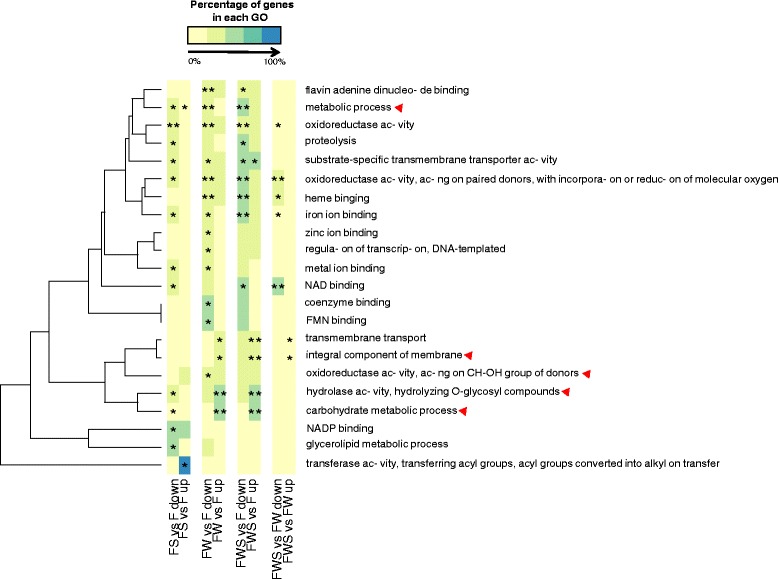
Functional enrichment of Gene Ontology (GO) categories based on the differentially expressed genes in each analyzed contrast. The color key indicate from pale *yellow* to *blue*, the increasing percentage of genes representing each functional category. To eliminate general and excessively specific categories, only the functional categories that have at least 5 and maximum 500 genes were considered in the analysis. The contrasting conditions evaluated are those indicated in Fig. 1. Briefly, FS/F: effect of heat stress (HS); FWS/FW: effect of HS but in the presence of grapevine wood; FWS/F: effect of HS in the presence of grapevine wood and FW/F: effect of only GW. * FDR < 0.2 and ** FDR < 0.05

On the other hand, the presence of GW resulted in repression of genes encoding enzymes with oxido-reductase activity (GO term: GO0016491) and induction of genes coding for O-glycosyl hydrolyzing enzymes (GO term: GO0004553, Fig. 4 and Fig. 5). In contrast, after HS, the expression pattern was inverse to the fungus growing in GW: genes for oxido-reductase activity were up-regulated while genes coding for O-glycosyl hydrolyzing enzymes were mainly down-regulated (Fig. 4 and Fig. 5). The genes involved in translation activities (GO terms: GO0008135 and GO0003743) and methyltransferase activity (GO term: GO0008168) showed primarily up-regulation in both contrasting conditions, FW/F and FWS/FW (Fig. 4), indicating the importance of the translation process in regulating the response to either change.

**Fig. 6:**
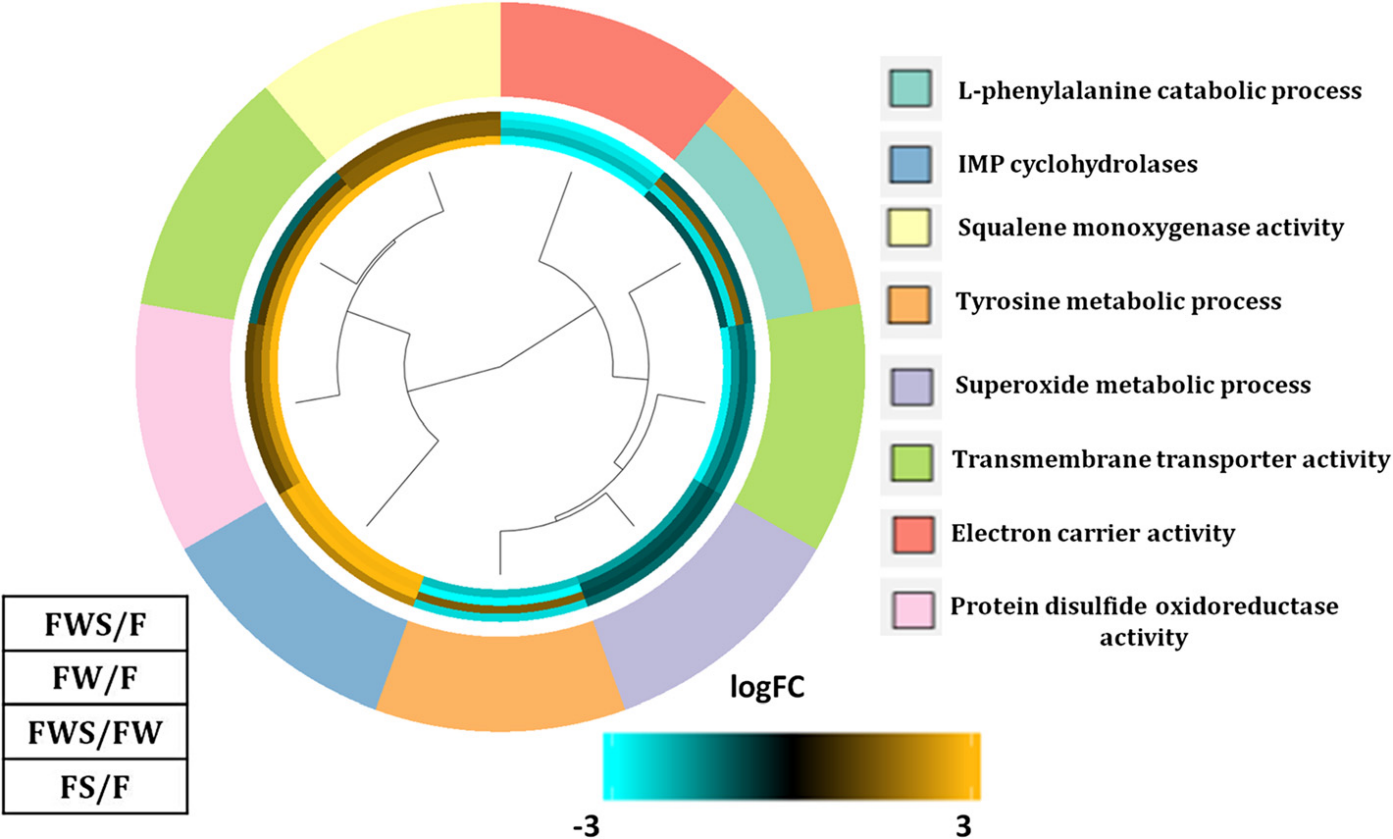
Gene Ontology (GO) of biological process categories with enrichment and showing differentially expressed genes in all the contrasting conditions evaluated (FDR < 0.05). The color key indicates logFC values ranging from golden for up-regulated genes, to cyan for down-regulated genes. The dendrogram indicates the relationship through the hierarchical clustering of gene expression (the middle rings show from the center out: effect on *L. theobromae* gene expression of heat stress (HS) and grapevine wood (FWS/F), only grapevine wood (FW/F), only HS (FS/F), and effect of HS in the presence of grapevine wood (FWS/FW). The Biological Process GOterms are indicated in different colors on the outermost circle

Our results showed that inosine monophosphate (IMP) cyclohydrolase, squalene monooxigenase and protein disulfide monooxigenase GO categories were induced in all the contrasting conditions (Fig. 6). According to the established functions of these GO categories, it seems that basic metabolism such as purine nucleotide biosynthesis, sterol biosynthesis and post-translational modifications are important both to sustain growth on GW and under HS. Similar basic mechanisms might be required to deal with both conditions, perhaps because oxidative stress is produced in response to HS, and also required for GW degradation. On the other hand, genes involved in tyrosine and L-phenylalanine metabolic processes were only induced in FWS/FW, while they were highly repressed in all other contrasted conditions, suggesting a specific requirement of this metabolic pathway to deal with HS when in contact with wood components (Fig. 6).

Secreted proteins are more prone to interact with the host; therefore we tested whether an enrichment of genes predicted to encode secreted proteins occurred among the DEGs. Among the upregulated genes in GW condition, there was an enrichment of those enconding for secreted proteins (FDR < 0.05 in Table 2), indicating that the presence of wood promotes the expression of secreted proteins. The list of genes coding for proteins presenting eukaryotic secretion signals, with their respective annotation, and classified in clusters based on co-regulation, is shown in Additional file 13.

### *L. theobromae* gene expression during the interaction with grapevine

Co-regulated clusters (Fig. 3) with functional enrichment indicated a whole fungal transcriptional change when exposed to HS in the presence of GW. Several genes belonging to any of the clusters 1, 3 and 7 (Fig. 3) that were identified as having pathogenicity roles, based on their functional annotation, were selected to evaluate their expression by RT-qPCR. cDNA obtained from in vitro conditions was used for the validation of RNAseq results, and cDNA from *in planta* conditions in an attempt to elucidate their role in pathogenicity.

The in vitro expression of 7 genes (Additional file 2: Table S1) confirmed the contrasting pattern of expression based on the cluster to which each gene belonged and showed a high correlation with the results obtained through RNAseq (R^2^ = 0.876 and p-value = 1.9x10^−5^, Additional file 14: Figure S6).

**Fig. 7:**
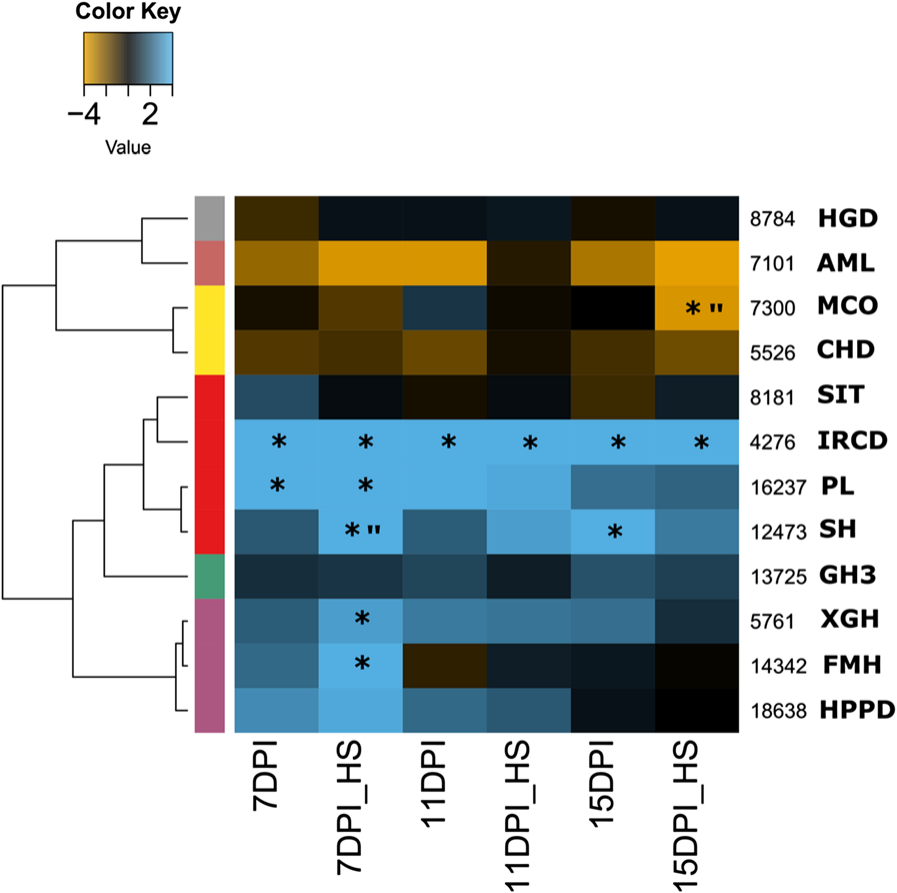
*L. theobromae* gene expression during grapevine infection. Fungal gene expression was evaluated at 7, 11 and 15 days post-infection in unstressed or heat-stressed grapevine (HS). Normalization was done using β-tubulin and the relative gene expression is indicated as logFC (*in planta*/in vitro) using HTqPCR package in R. One asterisk indicates significant differences with general linear model statistics (*p*-values < 0.05) for *in planta*/in vitro and quotation marks for heat-stressed/non stressed contrasting conditions. The dendrogram indicates the relationship between gene expression profiles through calculates of Canberra distances and Pearson correlation. The color key indicates logFC values ranging from golden for up-regulated genes, to cyan for down-regulated genes. IRCD: intradiol-ring cleavage dioxygenase (comp4276_c0_seq1); SH: salicylate hydroxylase (comp12473_c0_seq1); FMH: fumarylacetoacetate hydrolase (comp14342_c0_seq1); SIT:sugar inositol transporter (comp8181_c0_seq1), MCO: multicopper oxidase (comp7300_c0_seq1), PL: pectate lyase (comp16237_c0_seq1), AML: amylase (comp7101_c0_seq1), HGD: homogentisate dioxygenase (comp8784_c0_seq1), CHD: choline dehydrogenase (comp5526_c0_seq1), GH3: glycoside hydrolase family 3 (comp13725_c0_seq1), XGH: xylosidase glycoside hydrolase (comp5761_c0_seq2), HPPD: 4- Hydroxyphenylpyruvate dioxygenase (comp18638_c0_seq1)

In general when evaluating the expression *in planta*, a marked regulation of most genes was observed at 7 dpi, and the changes on gene expression were even more defined in stressed plants (Fig. 7). *L. theobromae* genes with up-regulated expression *in planta* were those enconding an intradiol ring cleavage dioxygenase (IRCD, comp4276_c0_seq1, selected from cluster 1), salicylate hydroxylase (SH, comp12473_c0_seq1, selected from cluster 1) pectate lyase (PL, comp16237_c0_seq1, selected from cluster 3), xylosidase glycoside hydrolase (XGH, comp5761_c0_seq2, selected from cluster 3) and a fumarylacetoacetate hydrolase (FMH, comp14342_c0_seq1, selected from cluster 1). IRCD was up-regulated at all infection times evaluated, in both heat-stressed and non-stressed plants (Fig. 6). At 7 dpi on stressed plants, SH was up-regulated when compared to infected and non-stressed grapevines (Fig. 7). In contrast, sugar inositol transporter (SIT, comp8181_c0_seq1), choline dehydrogenase (CHD, comp5526_c0_seq1), homogentisate dioxygenase (HGD, comp8784_c0_seq1), 4-hydroxyphenylpyruvate dioxygenase (HPPD, comp18638_c0_seq1) and glycoside hydrolase family 3 (GH3, comp13725_c0_seq1) did not show differential expression. A putative amylase from glycoside hydrolase family 35 (AML, comp7101_c0_seq1) and multicopper oxidase (MCO, comp7300_c0_seq1, from cluster 3) were highly down-regulated, specially under HS (Fig. 7).

## Discussion

### *L. theobromae* transcriptome features and comparison with related fungal pathogens

The closest fungi taxonomically related to *L. theobromae* with sequenced genomes are *M. phaseolina,* which has 14,249 genes (1,863 encoding secreted proteins (SP)) [57]*, D. seriata* with 9,398 genes (910 SP) and *N. parvum* with 10,470 genes (1,097 SP) [29]. The number of unique predicted proteins derived from unique isoforms (15,981), and those with predicted secretion signal (850) obtained through *de novo* transcriptome assembly, suggests that the number of genes in *L. theobromae* is similar to its closest relatives.

Most of the assembled contigs covered the expected transcript length (average: 1,310 bp and N50: 2,472 bp, details in Table 1), considering the 1,531 and 1,574 bp average gene length in *N. parvum* and *D. seriata*, respectively [21, 29]*.*

From the unique predicted proteins for all the transcripts, 399 had hits to PHI-base [47]. In *M. phaseolina*, 537 PHI-base hits were identified [57], while 1,120 were identified in *D. seriata* and 1,384 in *N. parvum* [29]. The differences found may be explained based on the different methods employed, since the relatives were fully sequenced and here RNAseq was done only in in vitro conditions. Further RNAseq analysis *in planta* will be useful to identify higher number of pathogenicity genes in this fungus. The putative pathogenicity transcripts (showing PHI-base hits) in *L. theobromae,* indicate main involvement in protein metabolic process, hydrolization of glycosyl bonds and oxidoreduction process (Fig. 2). In coincidence, these GO categories showed most marked regulation when the fungus growing on GW was exposed to HS (Fig. 4), suggesting that HS response and pathogenicity require a common transcriptional regulation. From the 399 PHI-base hits, 48 were differentially expressed, 22 were predicted to be secreted, and 8 of them shared both characteristics (Additional file 8), indicating that the *L. theobromae* transcriptome is useful to identify putative pathogenicity factors, that could help to further validation of function through knockout or gene expression studies.

Gene families showed expansion in fungi causing grapevine vascular diseases producing similar symptoms as *L. theobromae* [29]. Genes belonging to those families were found in the *L. theobromae* transcriptome, that have 65 (20 DEG) dioxygenase (PF00775), 11 (2 DEG) pectate lyase (PL, PF03211), 259 (49 DEG) major facilitator superfamily (MFS, PF07690), 37 (5 DEG) carboxylesterase (PF00135) and 22 (5 DEG) glucose-methanol-choline oxidoreductase (GMC, C-terminal: PF05199 and N-terminal: PF00732).

### Transcriptional regulation of genes with putative role in pathogenicity

Based on the GO enrichment by clusters containing co-regulated genes, a whole fungal response could be inferred (Fig. 4). In HS in the absence of GW, genes involved in the translation process were induced, suggesting that a change in protein profile is required to cope with stress. The requirement of heat-shock proteins (HSPs) in fungal HS response to keep proteins folded and active is well documented [58, 59]. In fact, several HSP encoding transcripts with different expression levels were identified, indicating the need of the fungal cells to redirect their physiology towards a survival or adaptive condition.

Based on functional enrichment, the fungus respond to HS differently in the presence of GW than in its absence. The specific enrichment of acyl-transferase among up-regulated genes when dealing with HS indicates that this activity could help the fungus to survive periods of stress. Acyl-transferases are involved in the synthesis of polyketides, a large class of secondary metabolites [60] that could provide the fungus with biochemical protection from stress. In contrast, in the presence of GW is not required, suggesting that some compounds, provided by GW fulfill that requirement. Genes in GO categories of integral component of membranes, mainly involved in transmembrane transport, were enriched specially in HS response in the presence of GW (Fig. 5). Most of these genes belong to the major facilitator superfamily (MFS) and have homologues on PHI base, suggesting an important role in fungal pathogenicity. Genes that belong to GO categories involved in nucleotide (GO:0050660), and post-translation modifications (Fig. 6) also are indicative of major change in the metabolism of the fungus to sustain growth under stress and in the presence of wood. On the other hand, GO term enriched GTPase activity, contains genes well known for having a role in translation and signaling events in response to stress [61], evidencing the importance of a rapid adaptation to HS. Additionally, small GTPases are involved in virulence, due to their key role in secretory pathways [61].

The repression of genes associated with electron transport (cytochrome p450 monooxygenase (comp5016_c0_seq3); dsba oxidoreductase protein (comp5774_c1_seq3), acyl- dehydrogenase family protein (comp7418_c0_seq1) and three hypothetical proteins (comp264619_c0_seq1, comp303165_c0_seq1 and comp1_c0_seq1), in response to HS and/or GW (Fig. 5), might contribute to the need of reducing the potential risk of mutation caused by higher production of ROS in mitochondria as an effect of thermal stress [62]. The down-regulation of a superoxide dismutase protein (comp6665_c0_seq1) is an unexpected result, because ROS detoxification has been proposed for superoxide dismutase and, increasing ROS levels in *Saccharomyces cerevisiae* were observed when responding to HS [63]. In the HS response in the absence of GW a 5-oxoprolinase (comp4986_c0_seq1) was specifically induced. This enzyme participate in glutathione mediated ROS detoxification [64], indicating that an alternative to superoxide dismutase/peroxidase could be used to deal with the ROS produced in response to HS.

**Fig. 8:**
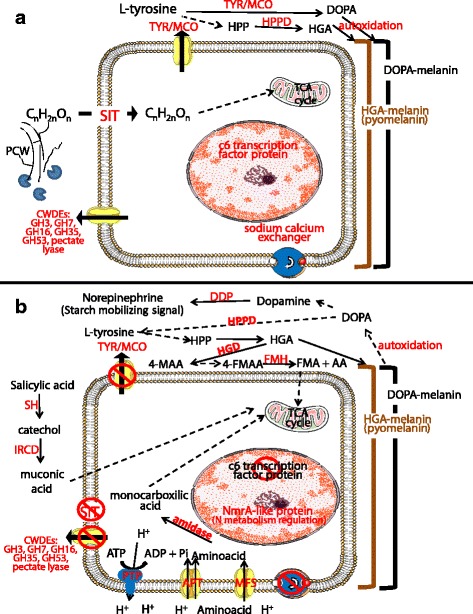
Hypothetical model of in vitro *L. theobromae* heat stress (HS) response, in the presence of grapevine wood (GW). The model of whole cell response was constructed using genes from clusters 1, 3 and 7 (in Fig. 2) showing both co-regulation and GO term enrichment. Additional file 13 gives the gene name with its expression pattern. The illustration shows the main functions and processes being carried out by the fungus when growing on GW (**a**), and the changes that are proposed to be occurring after HS (**b**). The putative cellular location (intra or extracellular) is indicated in the scheme. A *continuous arrow line* indicates an enzymatic reaction that is supported on the differentially expression of its encoding gene (shown in *red*). A *dashed arrow line* indicates a suggested enzymatic reaction. The substrate/product of reaction is indicated on *boxes*. TYR: tyrosinase, MCO: multicopper oxidase, DOPA: L-3,4-dihydroxyphenylalanine, HPP: 4-hydroxyphenylpyruvate, HPPD: 4-hydroxyphenylpyruvate dioxygenase, HGD: homogentisate dioxygenase, HGA: homogentisic acid, 4-MAA: 4-maleylacetoacetate, 4-FMAA: 4-fumarylacetoacetate, FM: fumarate, AA: acetoacetate, FMH:fumarylacetoacetate hydrolase, SH: salicylate hydroxylase, IRCD: intradiol ring cleavage dioxygenase, DDP: domon domain containing protein, PCWDEs: Plant cell wall degrading enzymes; PCW: Plant cell wall

A model for the *L. theobromae* HS response in the presence of GW was constructed (Fig. 8) based on the functional annotation, co-regulation and functional enrichment of genes (Additional file 15), that were identified through the in vitro global transcriptional response. Based on this model, several putative pathogenicity genes were selected and its gene expression was evaluated during fungal-grapevine interaction.

### Genes with putative role in grapevine wood degradation and pathogenicity

Most of the up-regulated genes in GW but down-regulated in HS are predicted to be involved in the degradation or modification of plant cell wall (PCW) components, based on their functional annotation (hydrolysis of O-glycosyl compounds, GO: 0004553, Fig. 4). A gene coding for a putative secreted enzyme that belongs to the glycosyl hydrolase (GH) family 35, has conserved domains which suggest its role as amylase (comp7101_c0_seq1). Its expression was induced by GW and showed no significant differences in the contrasting condition FWS/FW, indicating that degradation of starch remains active independently of HS. In grapevine wood, starch is stored in ray parenchyma cells, next to xylem vessels and it is used as carbon reserve by the plant [65]. *E. lata,* a vascular pathogen of grapevine that has similar colonization strategy as *L. theobromae*, has been shown to degrade plant starch [20] suggesting that this polymer may play a pivotal role as energy reserve for both the vascular pathogen and the plant metabolisms. Since the relationship among starch degradation and pathogenicity seems important for disease progression, the putative amylase (comp7101_c0_seq1) expression was evaluated *in planta.* Under stress conditions, it showed a marked down-regulation at any time of infection and treatment (Fig. 7), indicating that its activity is not required for the establishment of the infection, at least in the evaluated conditions. This enzyme was annotated as a putative amylase, because of one of its domain is a GH35; since this can be considered as an incomplete annotation, the enzyme encoded by comp7101_c0_seq1 sequence might have another function.

Among the genes coding for proteins predicted to be secreted, three are associated with pectin degradation: PL (comp16237_c0_seq1), GMC (comp7222_c0_seq1) and CHD (comp5526_c0_seq1). These genes encode for PCWDEs belonging to an expanded family in grapevine vascular pathogens [29]. The first one is involved in pectate cleavage [66], while the second and third one correspond to a gene family involved in lignin breakdown [67]. PL and CHD expression during fungal colonization was quite contrasting, while PL was significantly up-regulated, especially under HS at 7 dpi, CHD was down-regulated at any stage (Fig. 7). This suggests that lignin break-down was not required for fungal infection, but pectin degradation seems to be important for fungal pathogenicity. Pectin is the main component of middle lamella [68] and therefore have a fundamental role blocking intercellular fungal growth. Recently, it was clearly determined the importance of pectin degrading enzymes for *Fusarium graminearum* intercellular colonization of maize parenchyma tissue [69]. We propose that PL allows *L. theobromae* to colonize grapevine tissues intercellularly.

Genes encoding an endopolygalacturonase (comp4965_c2_seq1) and a pectinesterase (comp13568_c0_seq1) were also up-regulated in response to GW and have been associated with PCW degradation [65, 66]. Furthermore, expression of secreted enzymes, xylosidase glycoside hydrolase (XGH comp5761_c0_seq2), glycoside hydrolase family 3 (GH3 comp13725_c0_seq1) and endo1,4-betagalactanase (comp15147_c0_seq1), which are involved in cellulose and hemicellulose degradation [66], were found up-regulated in response to GW. Rolshausen et al. (2008) also reported in vitro hemicellulose degradation and increased enzymatic activity in the presence of wood in *E. lata* [20]. Two genes coding for enzymes that belong to GH1 with glucosidase/galactosidase activity (comp2943_c0_seq1 and comp7123_c0_seq1) have homologous genes with recognized intracellular roles in cellobiose and lactose catabolism [67]. The expression of both XGH and GH3 were up-regulated *in planta* (Fig. 7). XGH showed differential up-regulation at 7 dpi on infected and stressed plants (Fig. 7), suggesting that its function on hemicelluloses degradation is required, thus, deeper access to the host cell wall seems to be favored with HS.

**Fig. 9:**
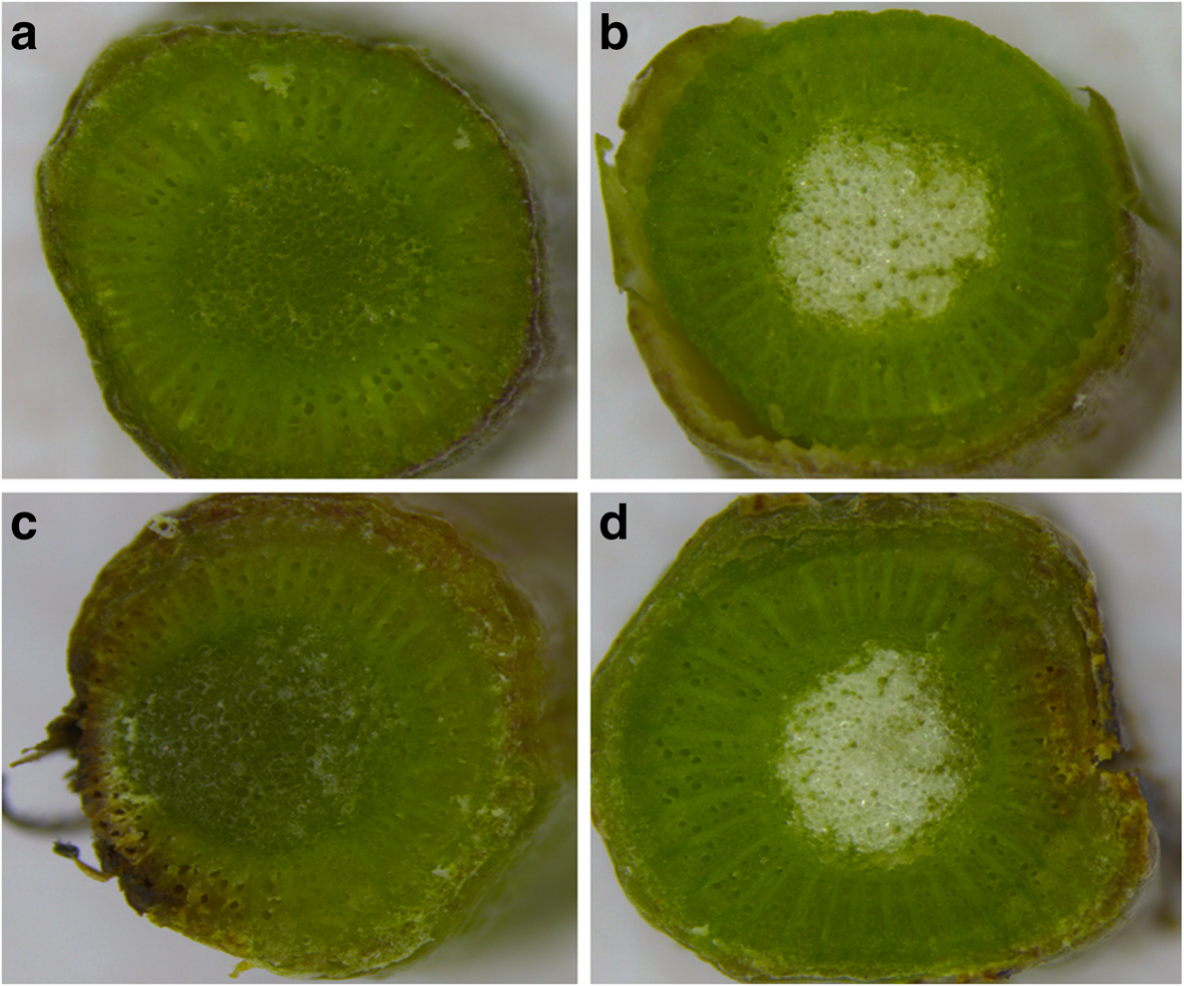
The effect of heat stress (HS) on grapevines uninfected or infected with *L. theobromae*. Stereomicroscope images were taken at 15 days post-infection, corresponding to 10 days of HS treatment (day-night cycles, with temperatures ranging from 42 to 20 °C). Uninfected and unstressed grapevine show healthy green tissue (**a**). Uninfected and heat-stressed grapevine show a change to a white color of the pith and phloem impairment (**b**). Infected but unstressed grapevine show *black*, *yellow* and *brown* color tissues close to the site of inoculation (**c**). Infected and stressed grapevine show *white pith* and *brown/yellow* color in primary xylem (**d**)

On other hand, an ortholog of the glyoxal oxidase (GOX) gene (comp11184_c0_seq1) showed up-regulation in the presence of GW. GOX was previously associated with PCW degradation of wood in *Phanerochaete carnosa*, specifically degrading lignin [70]. Furthermore, a gene (comp12572_c0_seq1) that belongs to GH16 encodes an enzyme involved in PCW modification [71] and showed up-regulation only in the presence of GW. Overall, the co-expression of these genes indicates polysaccharide degradation to use the GW as carbon-source. Its concerted enzymatic activity could help *L. theobromae* to colonize the host and cause the development of canker in wood. The necrosis observed close to the pith in heat-stressed plants (Fig. 9), likely indicates a deeper colonization of stem by *L. theobromae*. Our data suggest that the change in grapevine phenology occurs in response to HS and this change promotes a faster fungal colonization. Although not all the PCWDEs evaluated were up-regulated *in planta,* and even the putative amylase was down-regulated, it is possible that a specific role of up-regulated genes encoding PL and XGH favors fungal colonization under HS.

### Genes with putative role in phenolic, melanin, protein metabolism and pathogenicity

Several genes encoding enzymes involved in different melanin synthesis pathways reported in fungi were identified: 3,4-dihydroxyphenylalanine (DOPA)-melanin, DHN (1,8-dihydroxynaphthalene)-melanin and pyomelanin [72–78]. Remarkably, the gene for a secreted enzyme associated with DOPA-melanin synthesis (MCO, comp7300_c0_seq1) showed high induction in response to GW, while it did not show regulation upon HS (Additional file 15). This gene was found as belonging to a family expanded in *N. parvum* and *D. seriata*, stressing its putative role in pathogenicity [29]. A gene encoding enzyme putatively involved in DHN-melanin synthesis (short-chain dehydrogenase reductase, comp4978_c0_seq2) showed in vitro co-regulation with MCO. On the other hand, the enzyme involved in homogentisic acid synthesis, HPPD (comp18638_c0_seq1) was up-regulated (LFC = 2.18 and FDR = 0.07) in the presence of GW. This gene is associated with pyomelanin synthesis, a fungal adaptation of the L-tyrosine metabolic pathway, where the intermediary homogentisic acid is polymerized to form melanin [73, 74, 78].

After HS, genes that encode enzymes that degrade homogentisic acid, HGD (comp8784_c0_seq1) and FMH (comp14342_c0_seq1), were up-regulated, suggesting that pyomelanin degradation is activated after HS. The gene encoding maleylacetoacetate isomerase, involved in the transformation of 4-maleylacetoacetate to 4-fumarylacetoacetate in the L-tyrosine metabolic pathway [79], was identified (comp3928_c0_seq2 and comp196313_c0_seq1 in Additional file 16) but it was not differentially expressed in the analyzed contrasting conditions. The observation of L-tyrosine metabolic pathway regulation in response to HS is strengthened by the expression pattern (up-regulated by GW and down-regulated by HS) of the C6 transcription factor (comp6826_c0_seq1). This gene was previously found in the *Aspergillus fumigatus* genome, arranged in a cluster with the genes involved in gentisate catabolism [79], which is an alternative pathway to tyrosine metabolism in fungi. A mutant in the C6-Zn transcription factor *ProA* in *Epichloë festucae* was unable to establish a symbiotic interaction with its host *Lolium perenne*, proving the importance of this gene for the maintenance of the mutualistic relationship [80]. *L. theobromae* has an endophytic behavior [81, 82], and it has been suggested that abiotic stress promotes its switch to a pathogenic behavior [22, 83]. The C6 transcription factor regulating genes belonging to secondary metabolism pathways in response to HS, suggest the promotion of a pathogenic-like behavior in *L. theobromae* as an effect of HS.

Several yeast pathogens of mammals showed a phenotypic dimorphic switch (from filamentous to bud-cells and vice versa) when changing from room temperature to 37 °C, also accompanied with a change in melanin metabolism [84–87]. In general, a decrease in DOPA-melanin (tyrosinase downregulation) and/or an increase in pyomelanin production or L-tyrosine metabolism (4- hydroxyphenylpyruvate dioxygenase up-regulation) were observed consistently in previous studies [84–88]. It seems that in mammalian fungal pathogens, the change in the type of melanin produced is important to trigger the morphogenetic switch and to become pathogenic. We propose that a general mechanism for becoming pathogenic under HS conditions is conserved among several fungal taxa, whereby melanin metabolism could play a role in pathogenicity in plants, similarly to fungal mammalian pathogens. Our results also demonstrated that FMH was up-regulated *in planta* mainly at 7 dpi under HS (Fig. 7). A fumarylacetoacetase family was exclusively expanded in Ascomycota vascular pathogens [29], suggesting an evolutionary adapted function of these enzymes to favor the colonization of this niche. FMH is involved in the final step of the tyrosine metabolic pathway, converting fumarylacetoacetate to fumarate and acetoacetate, which could be incorporated in the tricarboxilyc acid cycle to obtain energy. Alternatively, the co-regulated genes, malate synthase (MS, comp6659_c0_seq1) and isocitrate lyase phosphorylmutase (ICP, comp8689_c0_seq1), suggest that acetate could be employed for anabolic processing through the glyoxylate cycle [89].

**Fig. 10:**
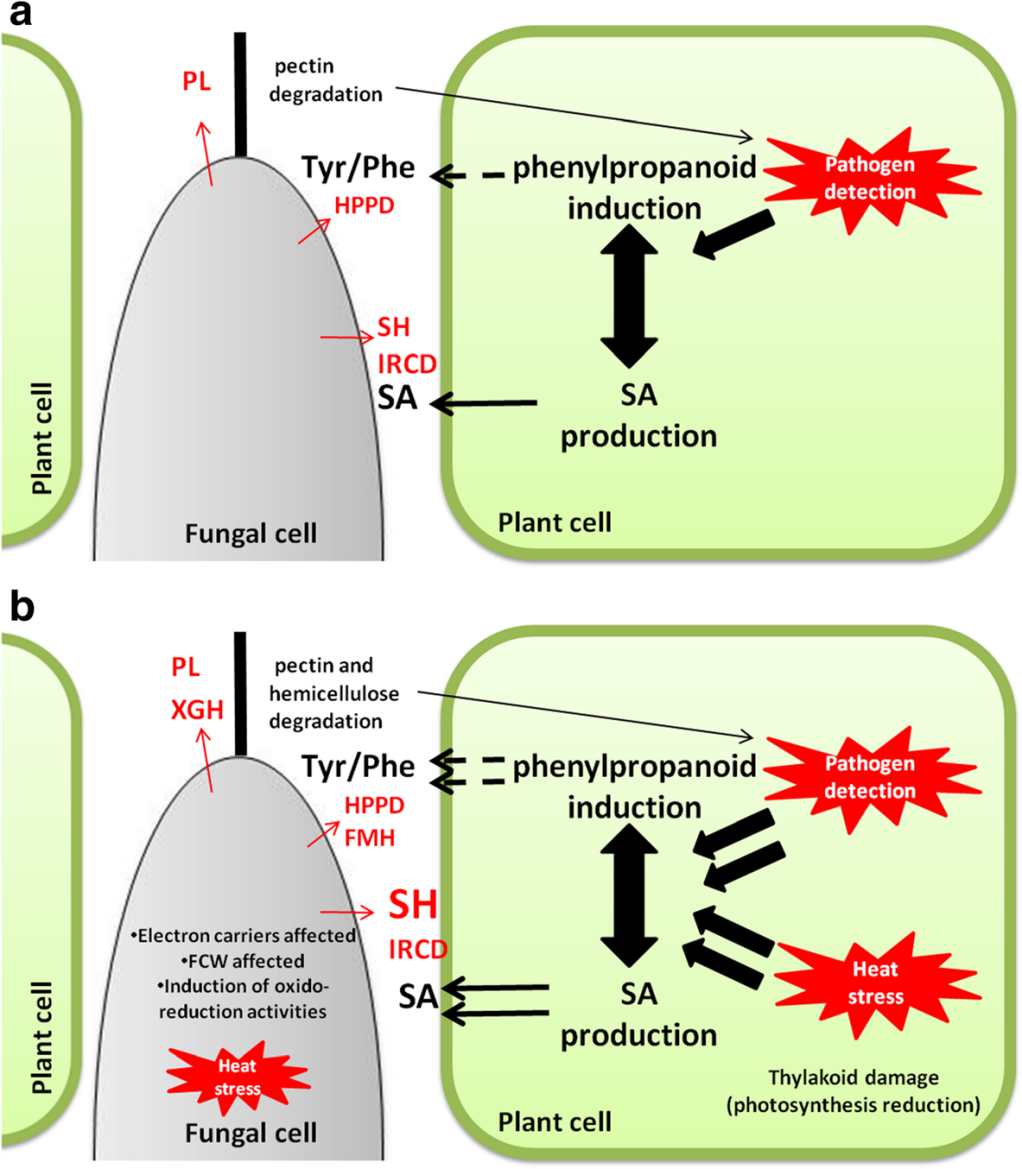
Proposed model of fungal-plant interaction under heat stress (HS). The model was done using gathered information of in vitro *L. theobromae* HS global transcriptional response, gene expression *in planta* (Fig. 7) and documented HS response in plant. **a** When fungus infects unstressed plant, selectively degrades pectin (up-regulation of PL compared with the other PCWDEs), this allows it to growth in intercellular spaces without causing major damage to PCW. The presence of the pathogen could trigger the host defensive mechanism mediated through SA, or by the activation of the phenylpropanoid pathway. However, the host defense could be impaired for the activity of SH and IRCD (degrade SA), or by HPPD (phenylpropanoid precursors). **b** Heat stress activates the same metabolic pathways increasing SA and the levels of phenylpropanoid precursors, thus the fungus could benefit from the higher availability of compounds using them as carbon sources. The main differential change on stressed plant, compared with unstressed, is the up-regulation of SH, FMH and XGH, whose activities could facilitate fungal colonization. Tyr: tyrosine. Phe: phenylalanine. SA: salicylic acid, IRCD: intradiol-ring cleavage dioxygenase (comp4276_c0_seq1); SH: salicylate hydroxylase (comp12473_c0_seq1); FMH: fumarylacetoacetate hydrolase (comp14342_c0_seq1); SIT:sugar inositol transporter (comp8181_c0_seq1); PL: pectate lyase (comp16237_c0_seq1). XGH: xylosidase glycoside hydrolase (comp5761_c0_seq2), HPPD: 4- Hydroxyphenylpyruvate dioxygenase (comp18638_c0_seq1). FCW: fungal cell wall. PCW: plant cell wall; PCWDEs: plant cell wall degrading enzymes

On other hand, it has been suggested that FMH regulates pyomelanin production [73, 74]. The homogentisate, an intermediary in L-tyrosine metabolic pathway, is used by some fungi as a precursor for pigment production [73, 74]. The precursors of lignin are derived from tyrosine or phenylalanine through the phenylpropanoid defensive pathway in plants [90]. This pathway is greatly induced in response to biotic and abiotic stress; therefore the fungal elimination of such precursors for DOPA-melanin or through tyrosine catabolic pathway could help the fungus to evade the plant compartmentalization mediated through lignin. When evaluated *in planta*, fungal MCO expression was down-regulated, specially under HS (Fig. 7). In contrast, HPPD and FMH were up-regulated at 7 dpi on stressed plants (Fig. 7), indicating that the fungal gene regulation under stress *in planta* is similar to that obtained in vitro (Fig. 7), supporting the hypothesis that DOPA-melanin could act as a tyrosine storage that then is degraded through tyrosine metabolism pathway. If this is the case, the increase of grapevine phenylpropanoid pathways precursors in response to HS, could be used by *L. theobromae* for its metabolism to promote colonization (Fig. 10).

In this work, SH (comp12473_c0_seq1) was up-regulated upon HS in the presence of GW during in vitro growth (Additional file 14: Figure S6). The product of SH activity, catechol, induces the ROS-mediated plant defensive response [91, 92]. IRCD (comp4276_c0_seq1), a gene coding for an enzyme that catalyze the degradation of aromatic rings [93], was co-regulated with SH. IRCD could act degrading catechol and therefore helping in salicylic acid (SA) degradation [94]. A plausible hypothesis is that the polymerization of catechol to melanin or its catabolism, could prevent this harmful compound from interacting with ROS activating enzymes. Despite the fact that benzoic acid (BZA) is used as a potent fungicide [95], *L. theobromae* growth was not inhibited in the presence of 15–30 mM of BZA (Additional file 17: Figure S7). No other carbon source was added to the medium VMM, suggesting that the fungus catabolizes BZA to sustain its growth. A marked increase in aerial mycelium and dark color was observed, indicating fungal melanin production [96–98] and revealing a relationship between phenolic degradation and melanin production.

Furthermore, we found that IRCD and SH expression in vitro were induced in response to HS when GW was present (Additional file 14: Figure S6). The reason for this response is not clear, since the fungus had no contact with a living plant. However, it suggests a conserved and advantageous response of the fungus to HS, since the enzyme is produced before the plant SA burst starts. To test this hypothesis, the fungal expression in infected grapevine was evaluated and both genes showed significant up-regulation at each time course evaluated (Fig. 7), therefore SH and IRCD could be required for grapevine colonization by the fungus. In particular, fungal SH showed a defined up-regulation when compared heat stressed to none stressed plants at 7dpi (Fig. 7). It was already reported that SA is produced in grapevine to alleviate the photosynthesis impairment produced under HS [99], suggesting that the fungus, instead of using carbohydrate-derived carbon, switches its metabolism to degrade one of the major and readily available plant hormones, i.e. SA (schematic model in Fig. 9). Indeed, in *Valsa mali-*apple interaction, the fungal benzoate pathway was enriched, indicating an important function in pathogenicity in a lignified host tissue [100]. SH was previously associated with *Ustilago maydis* pathogenicity promotion in maize, suggesting that SA is used as either a carbon source or to repress the plant defense response signal [101]. *Moniliophthora perniciosa,* a pathogen of cacao, contains a gene coding for SH in its genome, which could explain the high tolerance of this pathogen to SA [102]. Furthermore, SH was one of the most expressed genes during the endosymbiont phase of *E. festucae* in grass, suggesting a role for evading the host SA-mediated response [103]. We propose that SH and IRCD activities, help *L. theobromae* to disrupt the SA defensive system involved in Systemic Acquired Resistance, using at the same time SA as carbon source (Fig. 10).

A metalloproteinase (comp1851_c0_seq1) showed the most significant up-regulation in response to HS (Additional file 9). Metalloproteinases have previously been observed to be up-regulated in the necrotrophic stage of the pathogenic fungus *Moniliophthora roreri*-*Theobromae cacao* interaction [104] and belong to an expanded gene family in pathogenic Onygenales [105], indicating a putative role of this enzyme in pathogenicity.

On other hand, an NmrA-like protein-encoding gene (comp1446_c0_seq2) was down-regulated only in the presence of GW, but up-regulated with HS. NmrA is part of a system controlling nitrogen metabolite repression in fungi, acting as a negative transcriptional regulator involved in the post-translational modification of the transcription factor AreA [106–108]. The up-regulation of this gene upon HS, suggests a requirement for a different nitrogen source by fungal cells under thermal stress. Besides, AreA has been identified as an important regulator of secondary metabolism in *Fusarium fujikuroi* and *F. graminearum* [108, 109] and an NmrA-like protein was strongly up-regulated during a compatible stage in the *M. oryzae*-rice interaction [110], suggesting an important relationship between nitrogen metabolism and pathogenicity.

Other genes co-regulated with the NmrA-like protein and involved in nitrogen metabolism were a gene coding for an amino acid polyamine transporter I family (comp6363_c0_seq1) that is involved in amino acid/H+ transmembranal symport [111, 112] and several genes that belong to the MFS (comp23378_c0_seq1, comp7071_c0_seq1 and comp3035_c0_seq2) that are involved in general transport, including amino acid transport [113, 114]. The orthologs of genes encoding enzymes involved in L-tyrosine metabolism, HGD (comp8784_c0_seq1) and FMH (comp14342_c0_seq1), in *Penicillium marneffei*, showed regulation through AreA [77]. The above mentioned gene expression pattern suggests an important change in the general *L. theobromae* nitrogen metabolism as effect of HS.

## Conclusions

The transcriptome of *L. theobromae* was established, providing nucleotidic sequences to the scientific community that will help to understand and eventually control this important pathogen. Based on functional annotation, several genes with putative functions in pathogenicity were identified, highlighting those encoding PCWDEs and involved in phenolic metabolism. Genes involved in melanin production were co-regulated with key transcription factors that control carbon and nitrogen usage, suggesting that strong metabolic and morphological changes occurs during fungal heat stress adaptation, in a similar manner to those documented for some fungal mammal’s pathogens.

Based on the in vitro *L. theobromae* global transcriptional regulation under HS*,* it was possible to identify fungal genes with up-regulation both *in planta* under optimum conditions and also with distinctive expression under HS, which helped to establish a model of the fungal-plant interaction under stress. These results indicated that HS overall facilitated *L. theobromae* growth and colonization. We propose that these observations are triggered by the fact that phenylpropanoid precursors increase under heat stress, and that *L. theobromae* is able to utilize these compounds for its own metabolism Although additional experiments are required to evaluate the robustness of the proposed model, this work provides the foundation to future research in order to understand the complexity of this important grapevine vascular pathogen.

## Abbreviations

AML, putative amylase; BZA, benzoic acid; CHD, choline dehydrogenase; CTAB, cetyltrimethylammonium bromide; DEGs, differentially expressed genes; DEPC, dyethylpyrocarbonate; DHN; 1,8-dihydroxynaphthalene; DOPA, L-3,4-dihydroxyphenylalanine; dpi, days post-infection; FC, fold change; FDR, false discovery rate; FMH, fumarylacetoacetate hydrolase; GH, glycoside hydrolase; GH3, glycoside hydrolase family 3; GLM, General Linear Model; GMC, glucose-methanol-choline oxidorreductase; GO term, Gene Ontology functional term; GO, gene ontology; GW, grapevine wood; HGD, homogentisate dioxygenase; HPPD, 4- Hydroxyphenylpyruvate dioxygenase; HS, heat stress; HSP, heat-shock protein; ICP, isocitrate lyase phosphorylmutase; IMP, inosine monophosphate; IRCD, intradiol ring cleavage dioxygenase; MAI, maleylacetoacetate isomerase; MCO, multicopper oxidase; MFS, major facilitator superfamily; MS, malate synthase; NAP, nucleic acid preservation buffer; NCBI, National Center for Biotechnology Information; noHS, no heat stress; ORF, open reading frame; PCW, plant cell wall; PCWDEs, plant cell wall degrading enzymes; PDA, potato dextrose agar; PHI-base, Pathogen-Host interaction database; PL, pectate lyase; ROS, reactive oxygen species; RT-qPCR, reverse transcribed-quantitative PCR; SA, salicylic acid; SH, salicylate hydroxylase; SIT, sugar inositol transporter; TYR, tyrosinase; VMM, Vogel’s Minimal Medium; VS, Vogel’s salts; XGH, xylosidase glycoside hydrolase

## Additional files

Additional file 1: Figure S1. Pipeline employed in bioinformatics analysis. (TIF 1432 kb) Additional file 2: Table S1. List of primers employed in RT-qPCR. (XLSX 9 kb) Additional file 3: File S1. Fungal RNA extraction protocols. (PDF 496 kb) Additional file 4: Figure S2. Numbers and quality of reads obtained through Illumina Hiseq2500 sequencing (TIF 1437 kb) Additional file 5: Figure S3. Classification (Kingdom Fungi restringed) of Blastx hits according with the taxonomy information of orthologs. Transcripts were classified according with its taxonomy information through Blast2GO software (version 3.0, GO-DB version 2014–09). (TIF 1642 kb) Additional file 6: Figure S4. Main results of functional annotation in Blast2GO (version 3.0, GO-DB version 2014–09). (TIF 951 kb) Additional file 7: File S2. Classification of functional annotation based on Biological Process GO terms. (PDF 695 kb) Additional file 8: File S3. Functional annotation for transcripts that showed Blastp hits on Pathogen Host Interaction database, that were identified as differentially expressed and that present secretion signal on its encoding open reading frames. (XLSX 19 kb) Additional file 9: File S4. Statistical support of data distribution for differential gene expression quantification (PDF 620 kb) Additional file 10: File S5. List of genes with LFC and FDR values for each contrasting condition (XLSX 2599 kb) Additional file 11: Figure S5. Venn diagram indicating relations among differentially expressed genes among the contrast evaluated. Between brackets are shown the number of differentially expressed genes that were predicted to be secreted through SignalP 4.0 [41]. (PDF 1345 kb) Additional file 12: File S6. List of genes with functional annotation separated by clusters of co-regulated genes. (XLSX 216 kb) Additional file 13: File S7. List of genes with functional annotation and with secreted prediction separated by clusters of co-regulated genes. (XLSX 43 kb) Additional file 14: Figure S6. Differential expression quantification through RNA-seq and RT-qPCR. in vitro relative gene expression in FWS/FW (A) and FW/F (B) contrasting conditions calculated from RT-qPCR (light gray bars) and RNAseq data (dark gray bars). Normalization was done using β-tubulin for RT-qPCR data and count of reads per million (cpm) for RNAseq data. (C) Pearson correlation when comparing the LFC obtained with each technique. (TIF 2625 kb) Additional file 15: File S8. List of genes with functional annotation, LFC values and predicted protein sequence that were used to construct the model of *L. theobromae* response to HS (shown in Fig. 5). (XLSX 63 kb) Additional file 16: File S9. Raw blastx output of all transcripts (XLSX 16172 kb) Additional file 17: Figure S7.
*L. theobromae* growth on Vogel’s minimal medium (VMM) at 8 dpi amended with 0, 15 and 30 mM of Benzoic Acid (BZA). With increasing concentrations of BZA, higher density colonies with more aerial and darker mycelium were observed. (TIF 1718 kb)
