# Supplementary figures and images for: Global transcriptional analysis suggests Lasiodiplodia theobromae pathogenicity factors involved in modulation of grapevine defensive response
Source: BMC Genomics. 2016 Aug 11;17:615. doi: 10.1186/s12864-016-2952-3 (PMC4981995; doi:10.1186/s12864-016-2952-3)

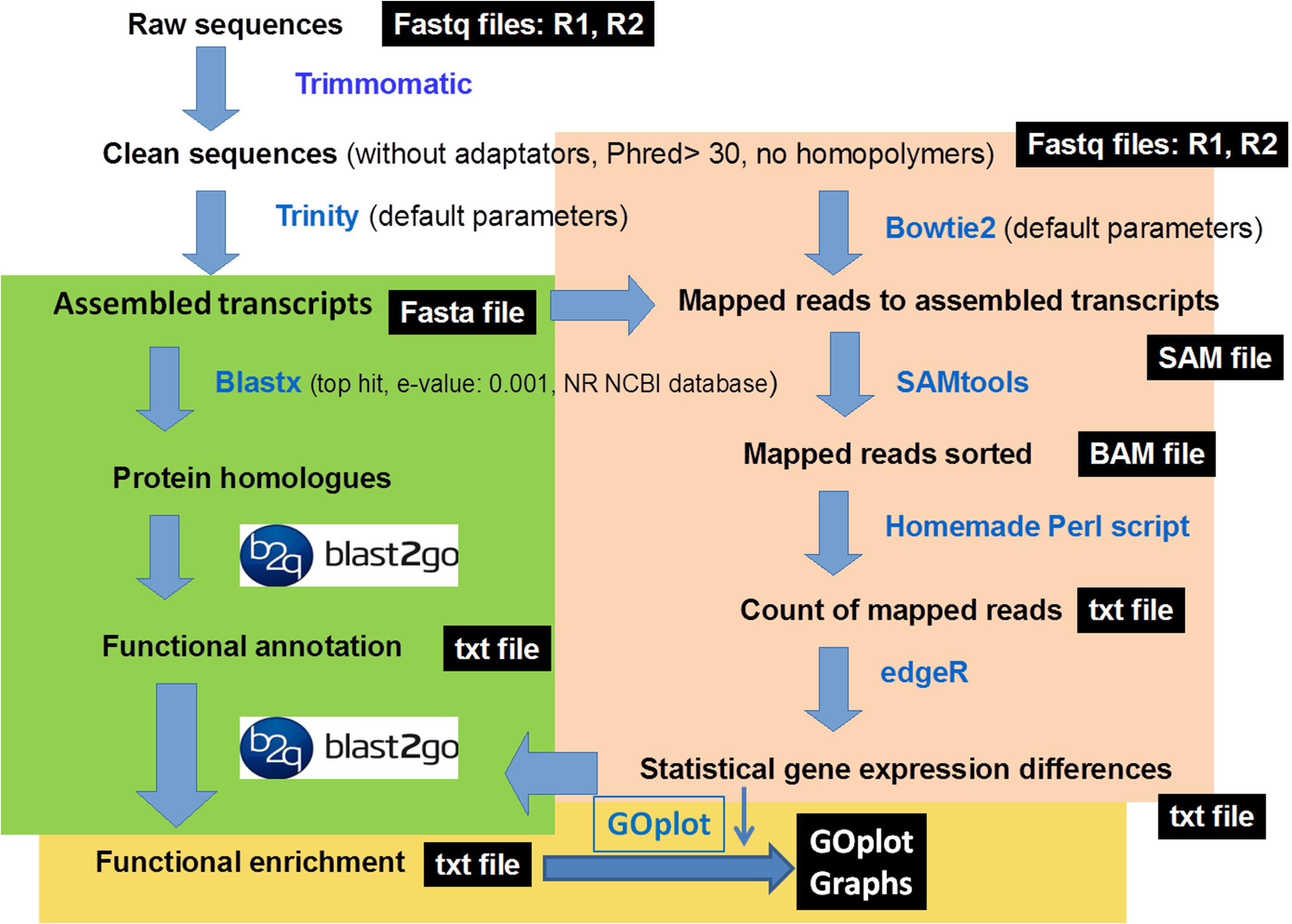

Supplement: Additional file 1: Figure S1. — Pipeline employed in bioinformatics analysis. (TIF 1432 kb) [file 12864_2016_2952_MOESM1_ESM.tif]

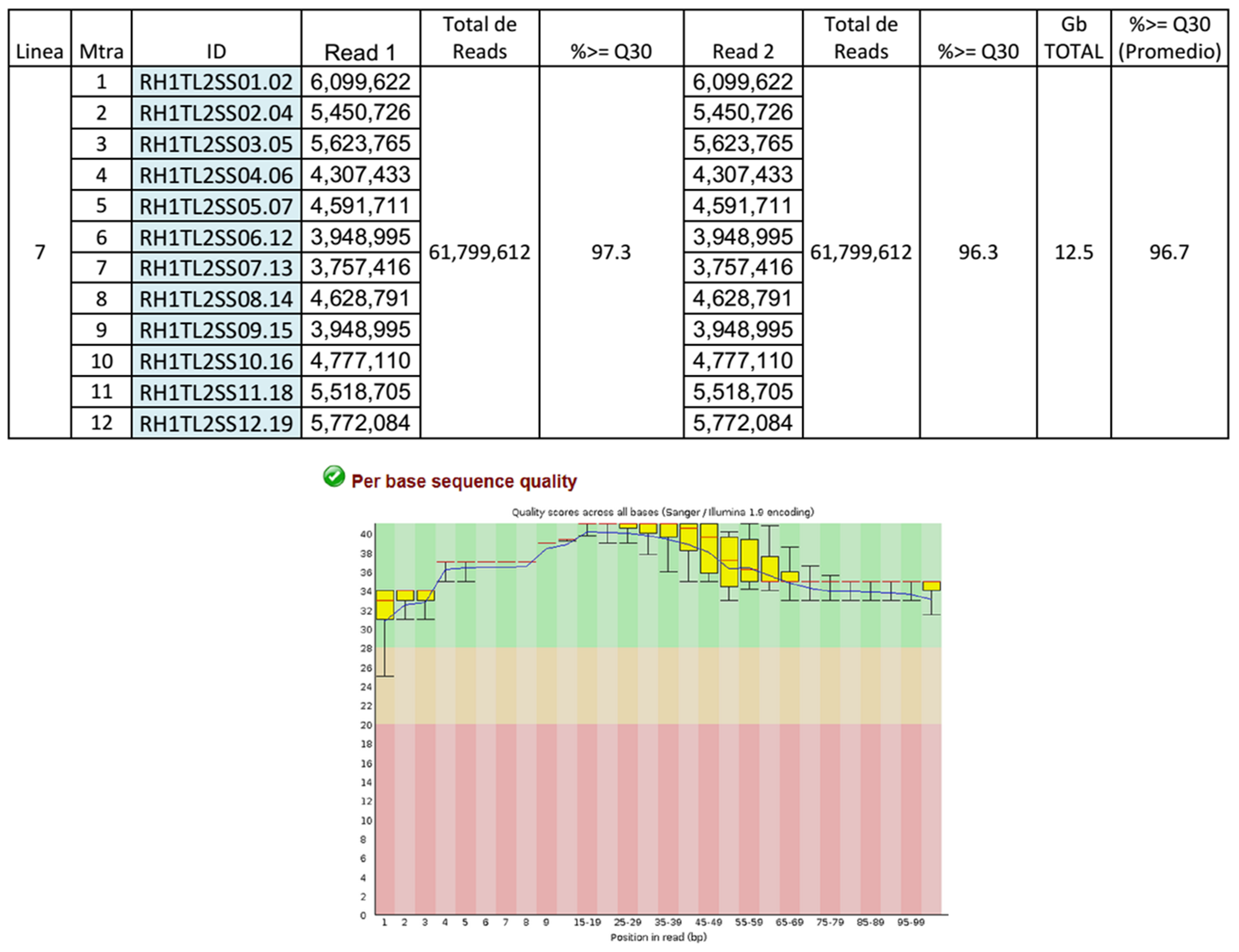

Supplement: Additional file 4: Figure S2. — Numbers and quality of reads obtained through Illumina Hiseq2500 sequencing (TIF 1437 kb) [file 12864_2016_2952_MOESM4_ESM.tif]

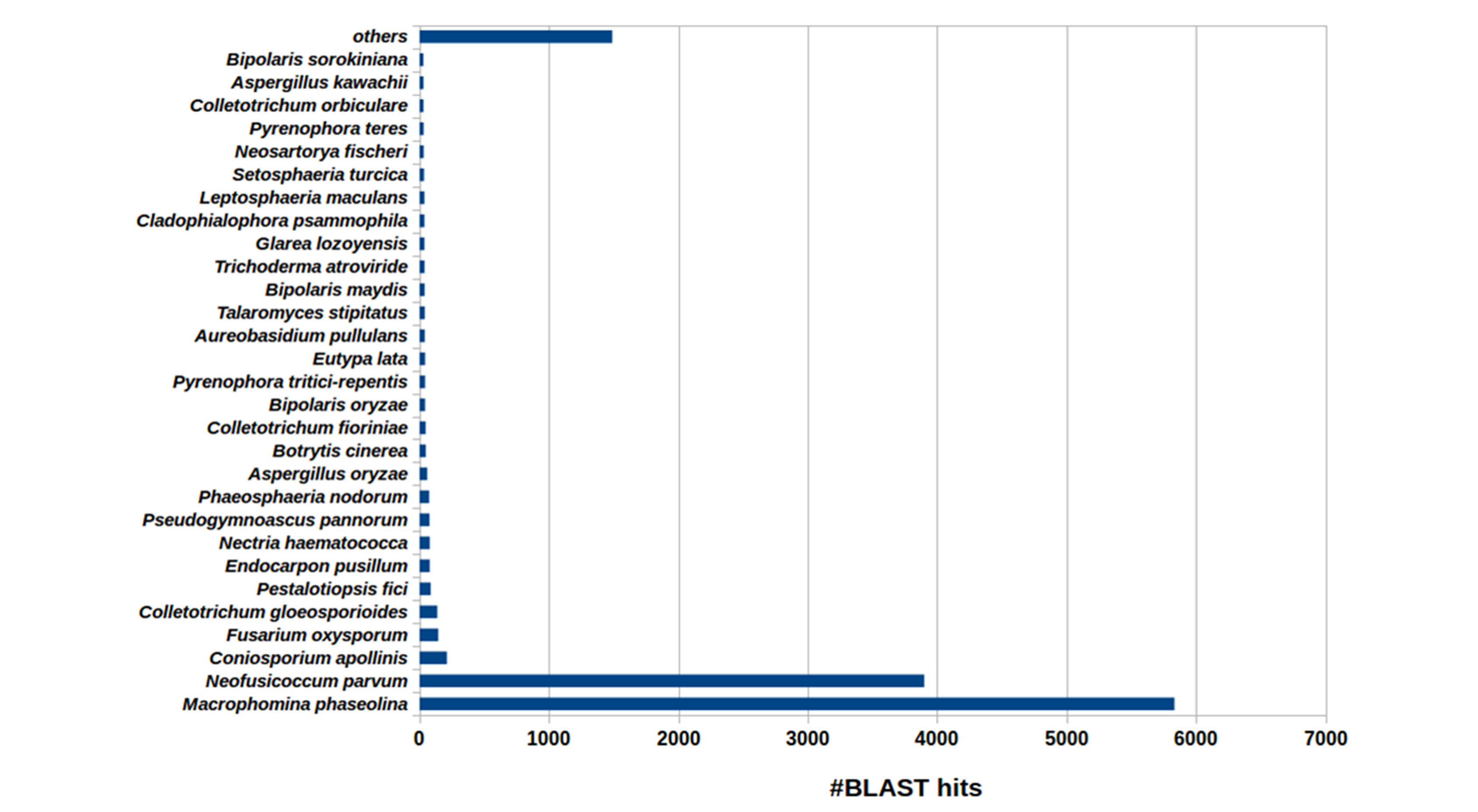

Supplement: Additional file 5: Figure S3. — Classification (Kingdom Fungi restringed) of Blastx hits according with the taxonomy information of orthologs. Transcripts were classified according with its taxonomy information through Blast2GO software (version 3.0, GO-DB version 2014–09). (TIF 1642 kb) [file 12864_2016_2952_MOESM5_ESM.tif]

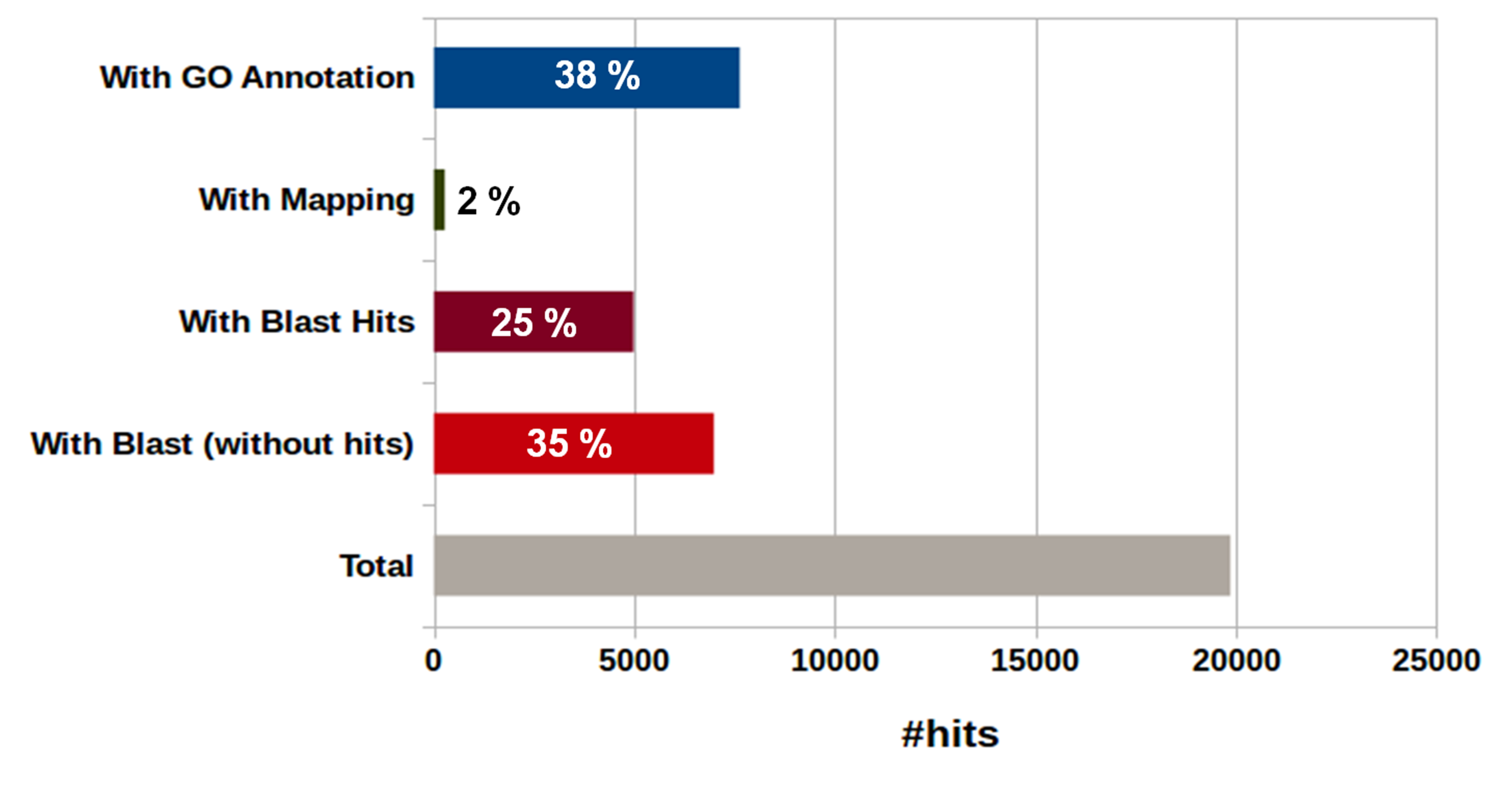

Supplement: Additional file 6: Figure S4. — Main results of functional annotation in Blast2GO (version 3.0, GO-DB version 2014–09). (TIF 951 kb) [file 12864_2016_2952_MOESM6_ESM.tif]

## UP-REGULATED

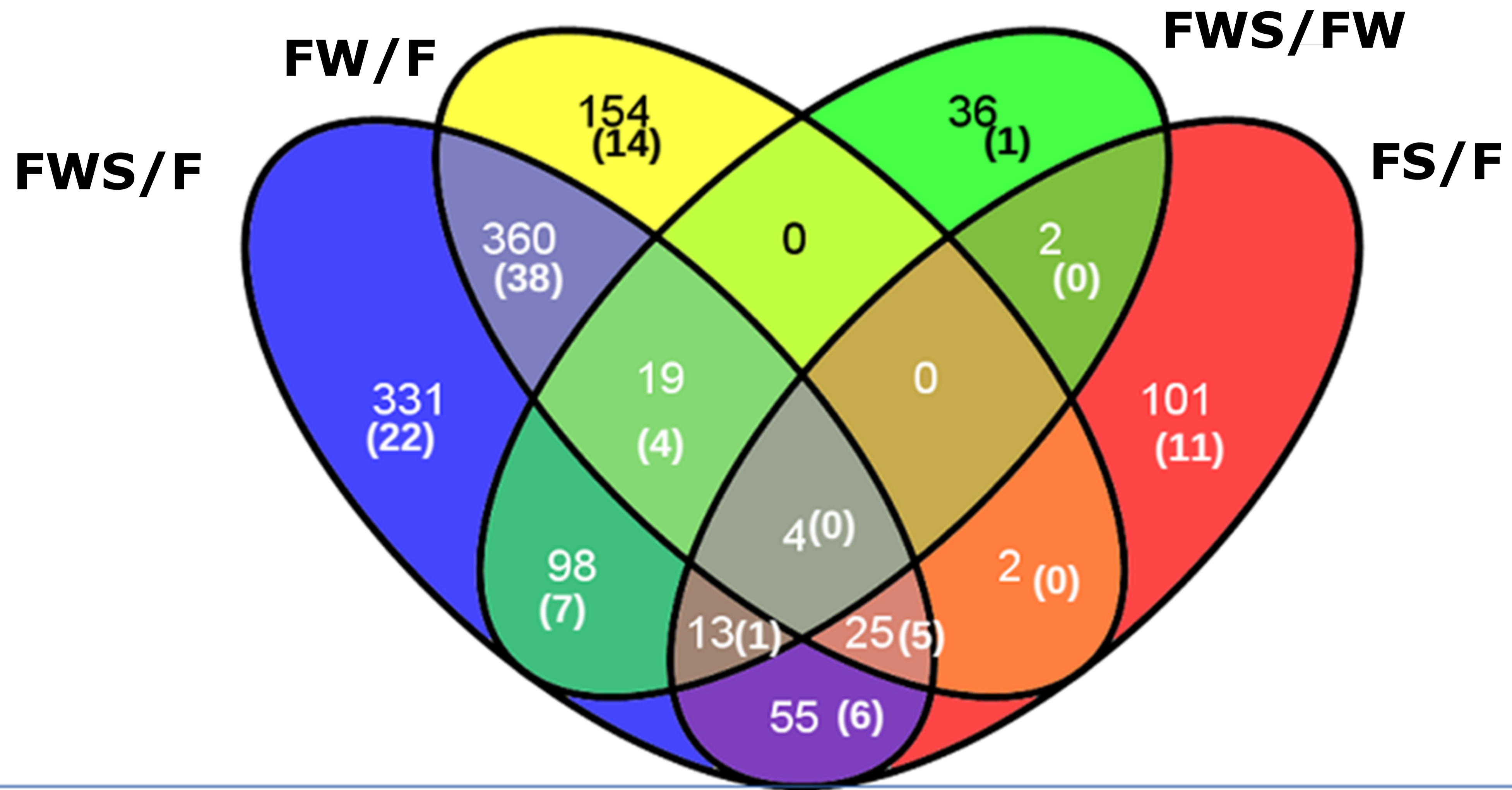

## DOWN-REGULATED

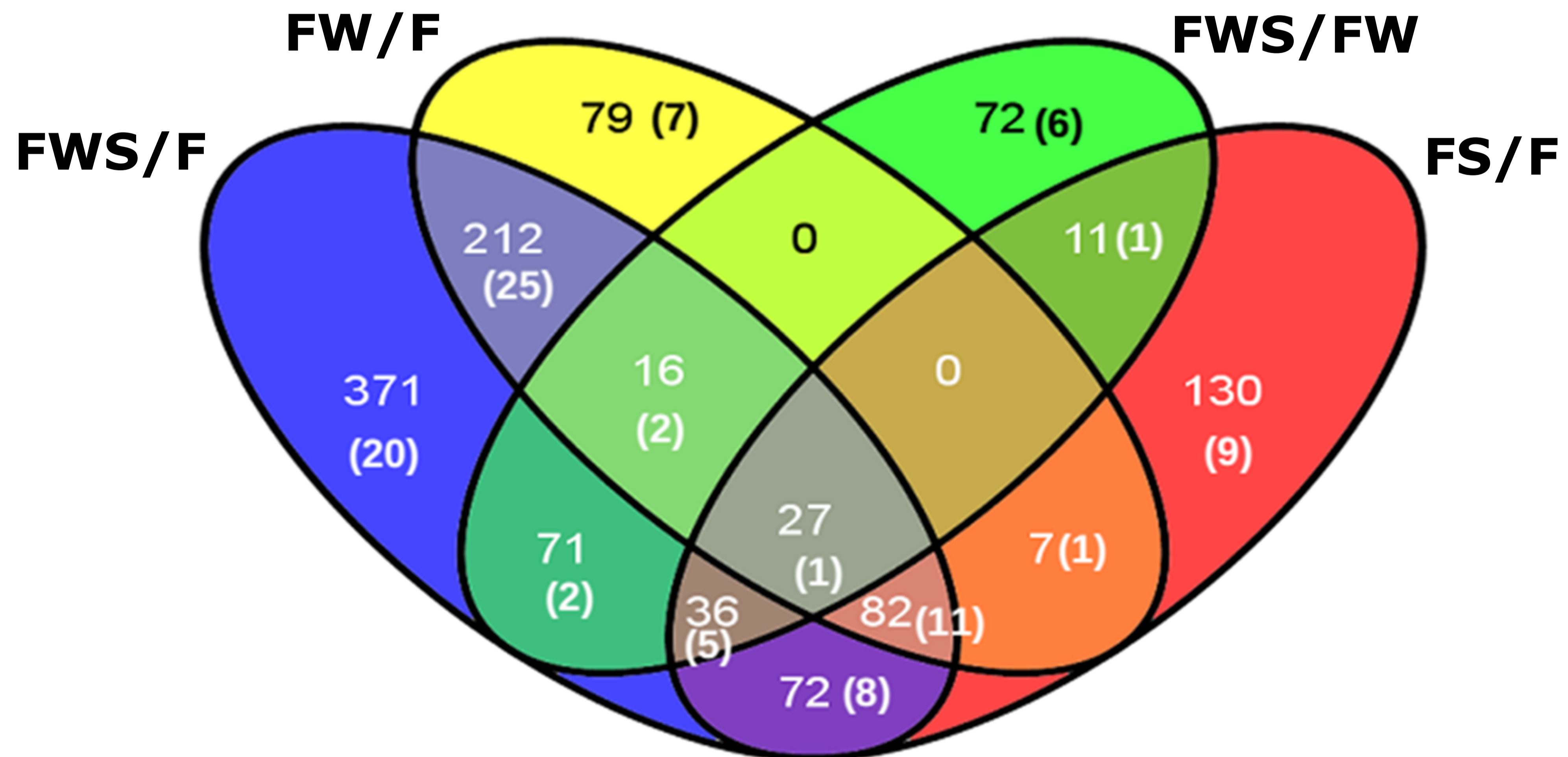

Supplement: Additional file 11: Figure S5. — Venn diagram indicating relations among differentially expressed genes among the contrast evaluated. Between brackets are shown the number of differentially expressed genes that were predicted to be secreted through SignalP 4.0 [41]. (PDF 1345 kb) [file 12864_2016_2952_MOESM11_ESM.pdf]

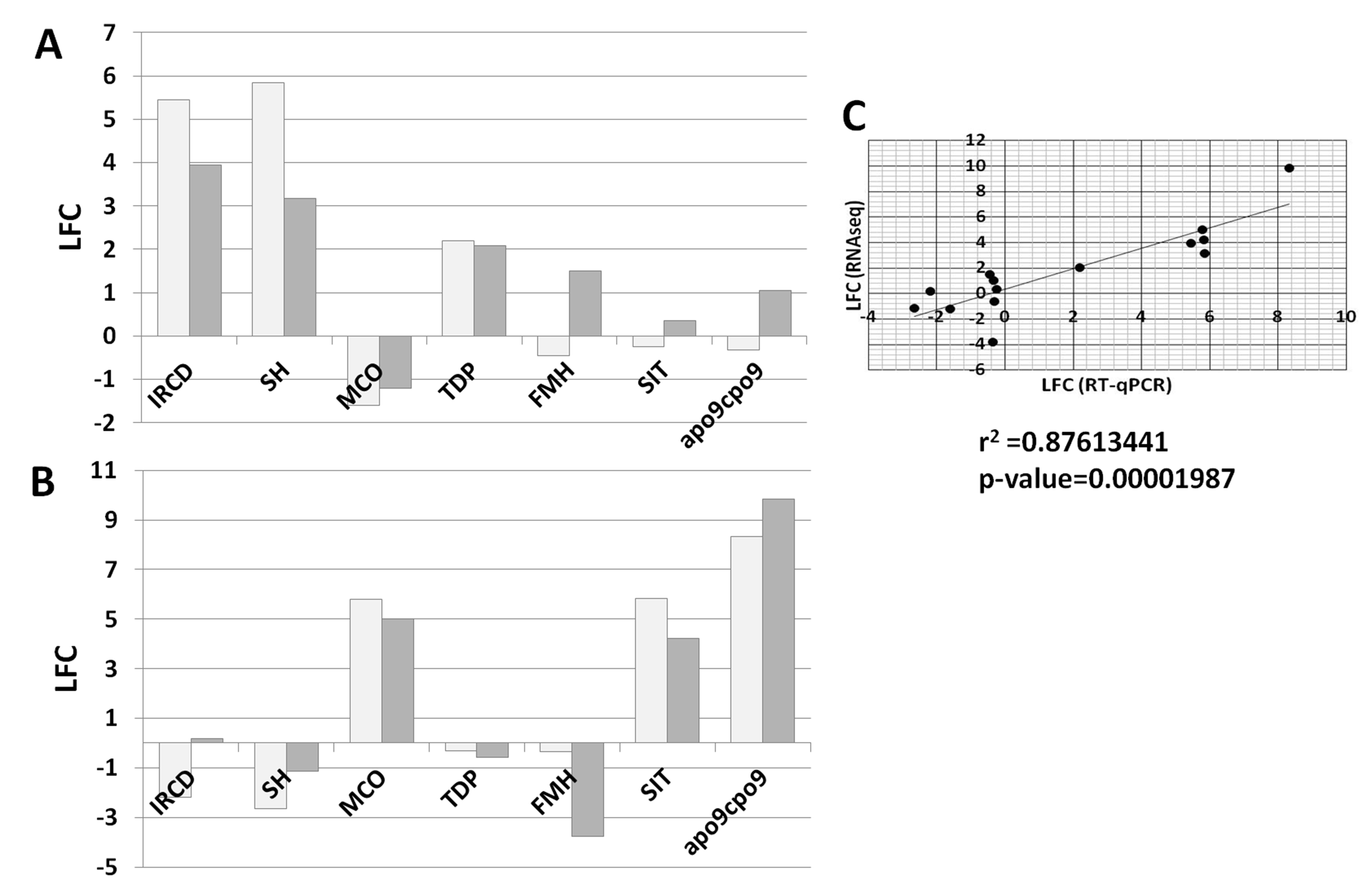

Supplement: Additional file 14: Figure S6. — Differential expression quantification through RNA-seq and RT-qPCR. in vitro relative gene expression in FWS/FW (A) and FW/F (B) contrasting conditions calculated from RT-qPCR (light gray bars) and RNAseq data (dark gray bars). Normalization was done using β-tubulin for RT-qPCR data and count of reads per million (cpm) for RNAseq data. (C) Pearson correlation when comparing the LFC obtained with each technique. (TIF 2625 kb) [file 12864_2016_2952_MOESM14_ESM.tif]

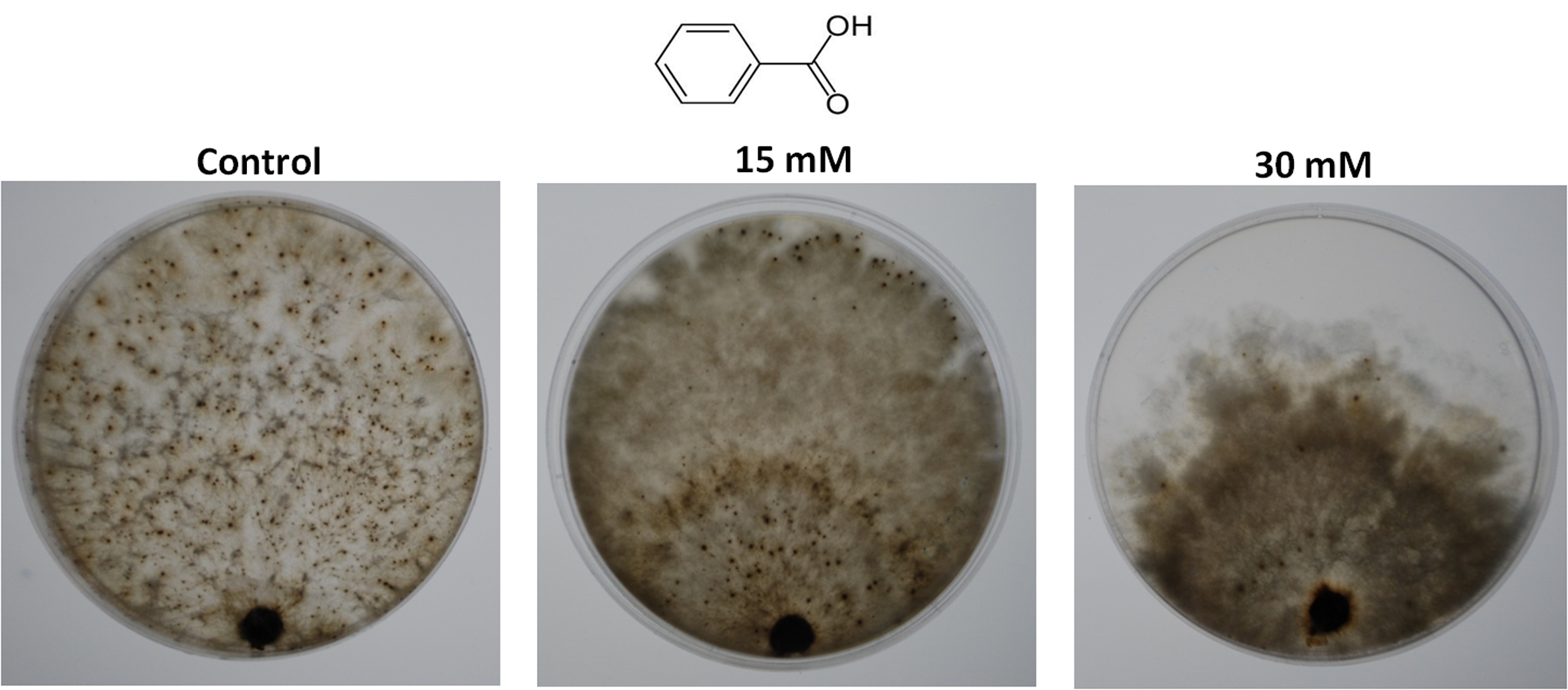

Supplement: Additional file 17: Figure S7. — L. theobromae growth on Vogel’s minimal medium (VMM) at 8 dpi amended with 0, 15 and 30 mM of Benzoic Acid (BZA). With increasing concentrations of BZA, higher density colonies with more aerial and darker mycelium were observed. (TIF 1718 kb) [file 12864_2016_2952_MOESM17_ESM.tif]
