# Supplementary material for: Global transcriptional analysis suggests Lasiodiplodia theobromae pathogenicity factors involved in modulation of grapevine defensive response
Source: BMC Genomics. 2016 Aug 11;17:615. doi: 10.1186/s12864-016-2952-3 (PMC4981995; doi:10.1186/s12864-016-2952-3)
Supplement: Additional file 3: File S1. — Fungal RNA extraction protocols. (PDF 496 kb) [file 12864_2016_2952_MOESM3_ESM.pdf]

File_S1 Global transcriptional analysis suggests *Lasiodiplodia theobromae* pathogenicity factors involved in modulation of grapevine defensive response

Marcos Paolinelli-Alfonso[1], Jose Manuel Villalobos-Escobedo[2], José Fabricio López-Hernández[2], Clara Galindo-Sánchez[3], Philippe Rolshausen[4], Alfredo Heriberto Herrera-Estrella[2] and Rufina Hernández-Martínez[1]

[1]Centro de Investigación Científica y de Educación Superior de Ensenada. Departamento de Microbiología. Ensenada, Baja California, México.[2]Laboratorio Nacional de Genómica para la Biodiversidad (LANGEBIO). Centro de Investigación y de Estudios Avanzados del I. P. N. Irapuato, Guanajuato, México.[3] Centro de Investigación Científica y de Educación Superior de Ensenada. Departamento de Biotecnología. Ensenada, Baja California, México.[4]Department of Botany and Plant Sciences, University of California Riverside, Riverside, CA 92521, USA.

RNA extraction from infected grapevine shoots

Total RNA from *L. theobromae*-infected grapevine was isolated using a cetyltrimethylammonium bromide (CTAB)-based extraction method (Gambino et al., 2008). Briefly, a day previous to RNA extraction, the transversal sections (around 5 mm of length and 100 mg of weight) of infected grapevines was removed from NAP solution and placed in 2.O mL tubes, containing one ¼ inch ceramic bead and 50 mg of 0.5 mm and 100 mg of 1 mm glass beads (Biospec). Tubes were beaten on a Mini-Beadbeater (Biospec Products) for 30 s. Immediately, tubes were chilled on ice, and pre-heated lysis solution was added (2% v/V CTAB, 2% v/V PVP, Tris-HCl 100 mM pH 8.0, NaCl 2 M, EDTA 25 mM, and 2% of β -mercaptoethanol added just before use) before subjecting the tubes to a second beating cycle for 30 s. Lysed samples were incubated at 65 °C for 5 min and 500 µl of CIA (24:1 v:v) was added and mixed twice in a Vortex. Tubes were centrifuged at 4,000 rpm for 30 min at 4 °C. The washing with CIA was repeated and then the aqueous phase was transferred to a new 1.5 mL tube and mixed with 1 v of 75% ethanol v:v. The total mix was passed through a silica column (Epochlab) and centrifuged at maximal velocity for 1 min at room temperature. Thereafter, the column was washed with RPE buffer (Qiagen) and twice with ethanol 75% v:v. Finally, total RNA was eluted in 48 µL of nuclease-free water (Qiagen) by centrifugation at maximal velocity.

References

Reid K.E., N. Olsson, J. Schlosser, F. Peng and S.T. Lund, 2006. An optimized grapevine RNA isolation procedure and statistical determination of reference genes for real-time RT-PCR during berry development. *BMC Plant Biology* 6: 27.

Vasanthaiah H.K.N., R. Katam and M.B. Sheikh, 2008. Efficient protocol for isolation of functional RNA from different grape tissue rich in polyphenols and polysaccharides for gene expression studies. *Electronic Journal of Biotechnology* 11: 1-9

Gambino, G., Perrone, I., and I. Gribaudo, 2008. A Rapid and effective method for RNA extraction from different tissues of grapevine and other woody plants. *Phytochemical Analysis : PCA*, *19*(6), 520–5. doi:10.1002/pca.1078
