# Supplementary material for: Global transcriptional analysis suggests Lasiodiplodia theobromae pathogenicity factors involved in modulation of grapevine defensive response
Source: BMC Genomics. 2016 Aug 11;17:615. doi: 10.1186/s12864-016-2952-3 (PMC4981995; doi:10.1186/s12864-016-2952-3)
Supplement: Additional file 7: File S2. — Classification of functional annotation based on Biological Process GO terms. (PDF 695 kb) [file 12864_2016_2952_MOESM7_ESM.pdf]

File_S2 Global transcriptional analysis suggests *Lasiodiplodia theobromae* pathogenicity factors involved in modulation of grapevine defensive response

Marcos Paolinelli-Alfonso[1], Jose Manuel Villalobos-Escobedo[2], José Fabricio López-Hernández[2], Clara Galindo-Sánchez[3], Philippe Rolshausen[4], Alfredo Herrera-Estrella[2] and Rufina Hernández-Martínez[1]

[1]Centro de Investigación Científica y de Educación Superior de Ensenada. Departamento de Microbiología. Ensenada, Baja California, México.[2]Laboratorio Nacional de Genómica para la Biodiversidad (LANGEBIO). Centro de Investigación y de Estudios Avanzados del I. P. N. Irapuato, Guanajuato, México.[3] Centro de Investigación Científica y de Educación Superior de Ensenada. Departamento de Biotecnología. Ensenada, Baja California, México.[4]Department of Botany and Plant Sciences, University of California Riverside, Riverside, CA 92521, USA.

The functional annotation was done through Blast2GO using all the sequences assembled of transcripts (all the conditions together). The gene ontology structure employs several levels to classify the relations among GOterms (Ashburner et al., 2000) using higher numbers for more specific categories. The three trunk classification was molecular function, biological process or cellular component (Ashburner et al., 2000).

Figure 1. Biological process in second level of gene ontology classification. Most of the transcripts are involved in metabolic processes (2,283) and cellular processes (1,750). Also a good number are classified as single-organism processes (801) and localization (607). And with a significant number also are represented process like biological regulation (351), response to stimulus (176) and signaling (87).

Figure 2. Biological process in third level of gene ontology classification. Most of the transcripts were distributed in primary metabolic processes (1316), organic substances metabolic process (1405), nitrogen compound metabolic processes (796), establishment of localization (603), single organism cellular (729) and metabolic (954) processes.

Figure 3. Biological process in fourth level of gene ontology classification. Most of the transcripts were involved in nitrogen metabolism (684), aromatic (665), organic cycles (689) and heterocycle (676) compounds metabolism. Also there are 589 transcripts involved in oxidation-reduction process.

Figure 4. **Biological process in fifth level of gene ontology classification.** Most of the transcripts were involved in cellular macromolecule metabolic process (103), nucleobase-containing compound metabolic process (66), gene expression (65) and macromolecule biosynthetic process (65).

Fig 1

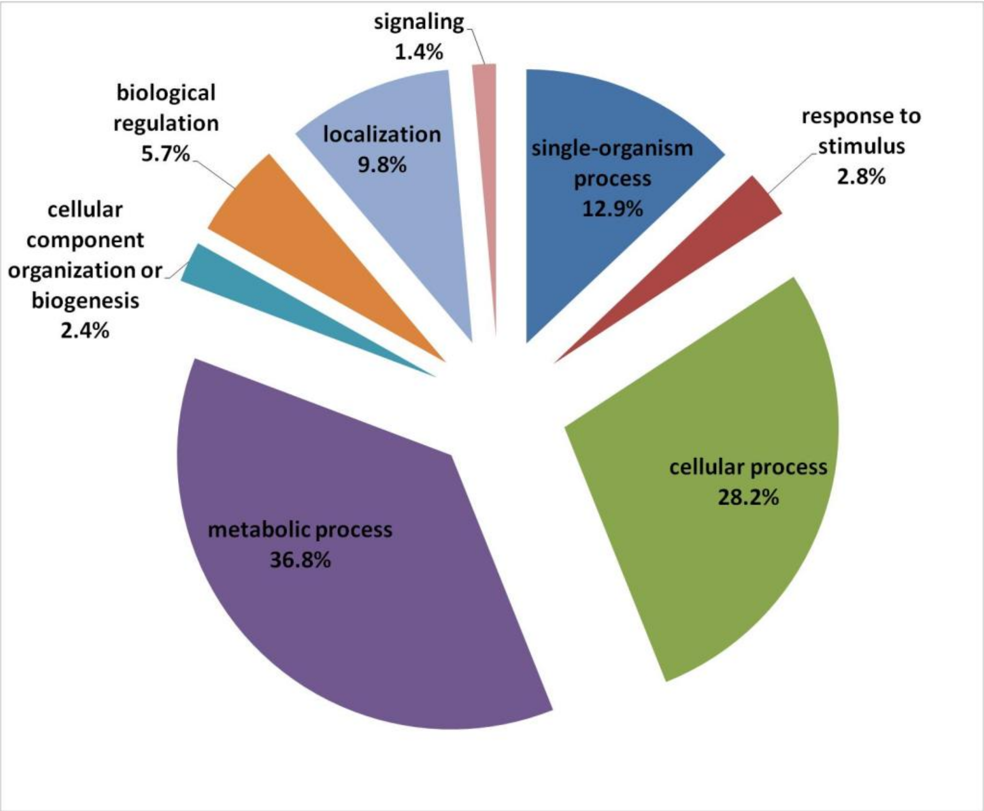
signaling
1.4%
response to
stimulus
2.8%
biological
regulation
5.7%
localization
9.8%
single-organism
process
12.9%
cellular
component
organization or
biogenesis
2.4%
cellular process
28.2%
metabolic process
36.8%

Fig 2

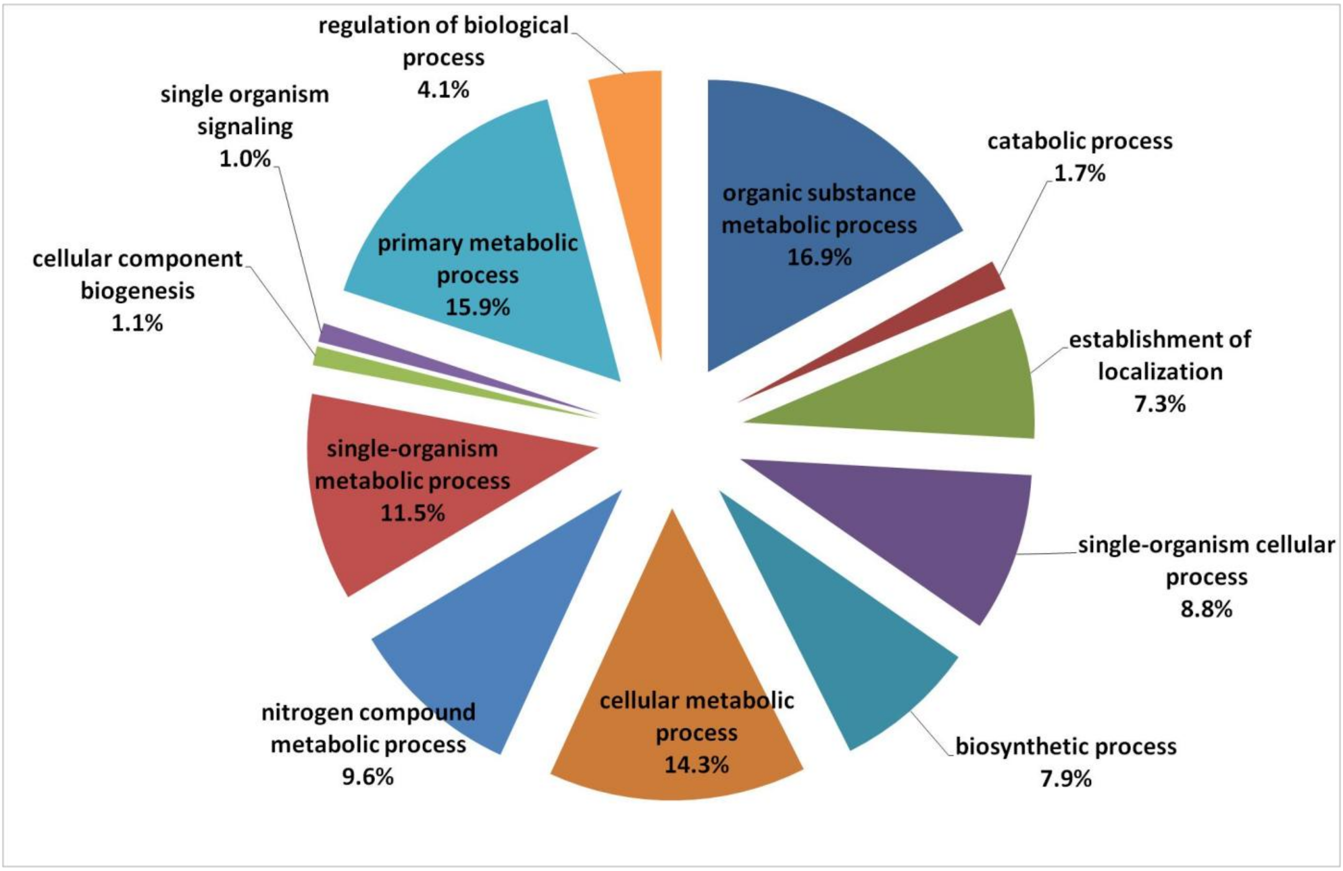
regulation of biological process 4.1%
single organism signaling 1.0%
cellular component biogenesis 1.1%
primary metabolic process 15.9%
organic substance metabolic process 16.9%
catabolic process 1.7%
establishment of localization 7.3%
single-organism metabolic process 11.5%
single-organism cellular process 8.8%
nitrogen compound metabolic process 9.6%
cellular metabolic process 14.3%
biosynthetic process 7.9%

Fig 3.

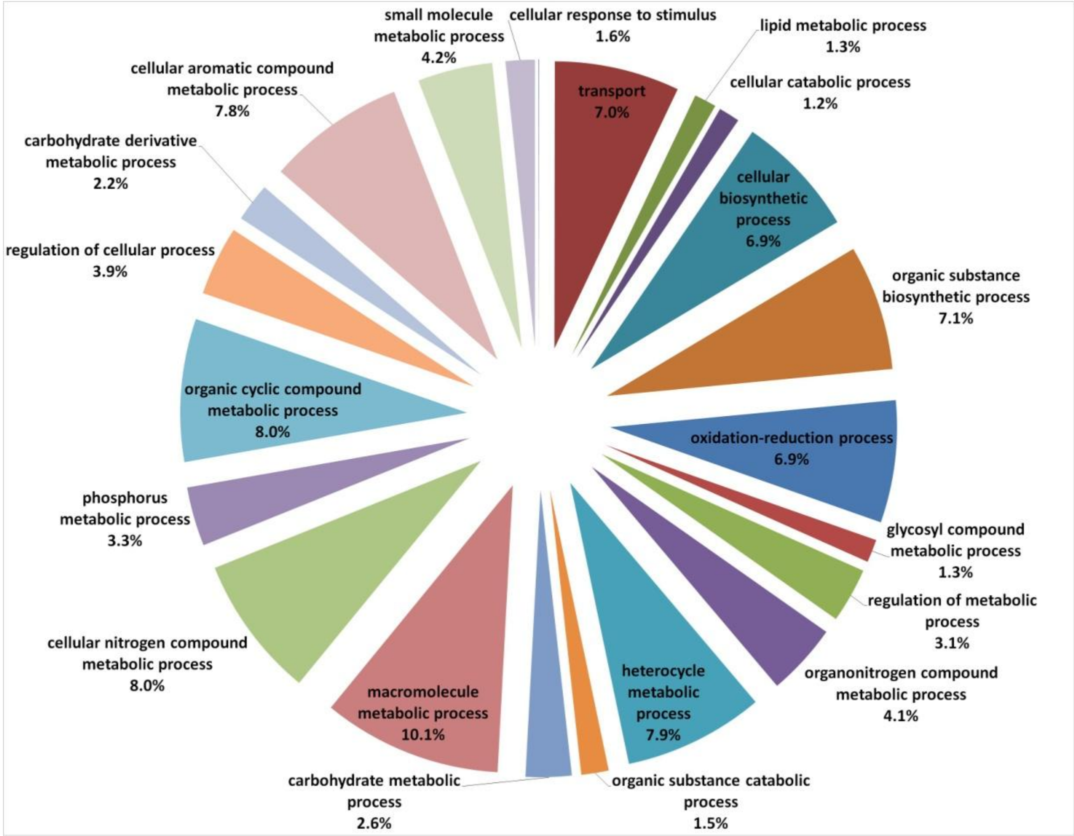
small molecule metabolic process 4.2%
cellular response to stimulus 1.6%
lipid metabolic process 1.3%
cellular catabolic process 1.2%
transport 7.0%
cellular aromatic compound metabolic process 7.8%
carbohydrate derivative metabolic process 2.2%
cellular biosynthetic process 6.9%
regulation of cellular process 3.9%
organic substance biosynthetic process 7.1%
organic cyclic compound metabolic process 8.0%
oxidation-reduction process 6.9%
phosphorus metabolic process 3.3%
glycosyl compound metabolic process 1.3%
regulation of metabolic process 3.1%
cellular nitrogen compound metabolic process 8.0%
heterocycle metabolic process 7.9%
organonitrogen compound metabolic process 4.1%
macromolecule metabolic process 10.1%
carbohydrate metabolic process 2.6%
organic substance catabolic process 1.5%

Fig 4

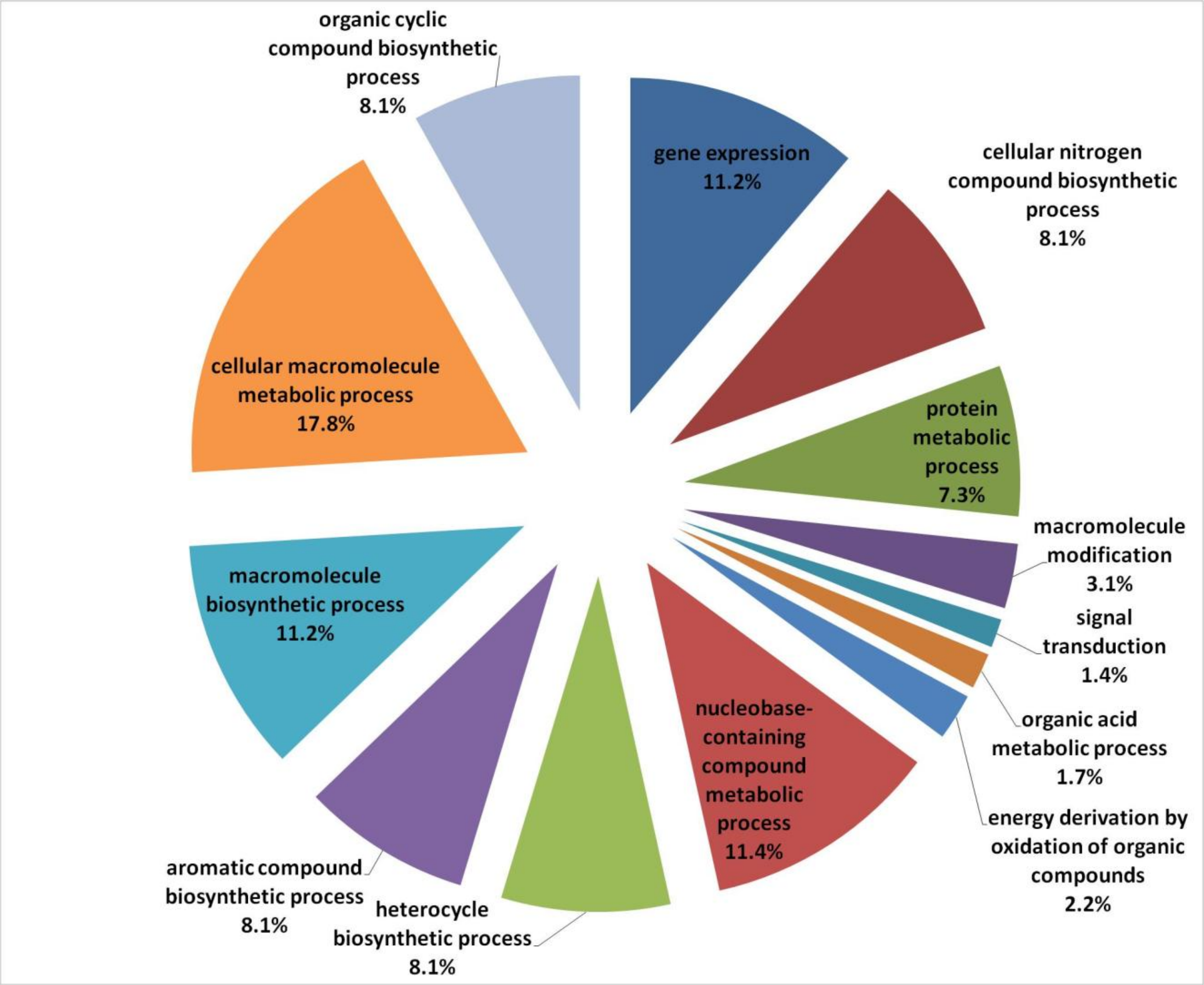
organic cyclic compound biosynthetic process 8.1%
gene expression 11.2%
cellular nitrogen compound biosynthetic process 8.1%
protein metabolic process 7.3%
macromolecule modification 3.1%
signal transduction 1.4%
organic acid metabolic process 1.7%
energy derivation by oxidation of organic compounds 2.2%
nucleobase-containing compound metabolic process 11.4%
heterocycle biosynthetic process 8.1%
aromatic compound biosynthetic process 8.1%
macromolecule biosynthetic process 11.2%
cellular macromolecule metabolic process 17.8%

References

Ashburner, M., Ball, C. A., Blake, J. A., Botstein, D., Butler, H., Cherry, J. M., … Sherlock, G. (2000). Gene ontology: tool for the unification of biology. The Gene Ontology Consortium. *Nature Genetics, 25*(1), 25–9. doi:10.1038/75556
