# Supplementary material for: Global transcriptional analysis suggests Lasiodiplodia theobromae pathogenicity factors involved in modulation of grapevine defensive response
Source: BMC Genomics. 2016 Aug 11;17:615. doi: 10.1186/s12864-016-2952-3 (PMC4981995; doi:10.1186/s12864-016-2952-3)
Supplement: Additional file 9: File S4. — Statistical support of data distribution for differential gene expression quantification (PDF 620 kb) [file 12864_2016_2952_MOESM9_ESM.pdf]

File_S4 Global transcriptional analysis suggests *Lasiodiplodia theobromae* pathogenicity factors involved in modulation of grapevine defensive response

Marcos Paolinelli-Alfonso[1], Jose Manuel Villalobos-Escobedo[2], José Fabricio López-Hernández[2], Clara Galindo-Sánchez[3], Philippe Rolshausen[4], Alfredo Heriberto Herrera-Estrella[2] and Rufina Hernández-Martínez[1]

[1]Centro de Investigación Científica y de Educación Superior de Ensenada. Departamento de Microbiología. Ensenada, Baja California, México.[2]Laboratorio Nacional de Genómica para la Biodiversidad (LANGEBIO). Centro de Investigación y de Estudios Avanzados del I. P. N. Irapuato, Guanajuato, México.[3] Centro de Investigación Científica y de Educación Superior de Ensenada. Departamento de Biotecnología. Ensenada, Baja California, México.[4]Department of Botany and Plant Sciences, University of California Riverside, Riverside, CA 92521, USA.

Figure 1. Biological coefficient of variation (BCV) of normalized data obtained using the plotBCV function in edger package version 3.4.2 (Robinson et al., 2010). The square root of common BCV is denoted by the red line. CPM: counts per million.

Figure 2. Two-dimensional scanning plot of normalized data obtained using plotMDS function in edgeR edgeR package version 3.4.2 (Robinson et al., 2010) (A) and a three-dimensional scanning plot (B) obtained through plot3d function from the library rgl version 0.93.996 (Adler et al. 2014, http://CRAN.R-project.org/package=rgl).

Figure 3. Dispersion of normalized data analyzed through Smear plot (left panel) and Volcano plot (right panel) for contrasting conditions FWS/FW (A), FWS/F (B), FW/F (C) and FS/F (D). Red dots indicate differentially expressed genes. FC: Fold change. FDR: False Discovery Rate. Graphs were obtained with edgeR package version 3.4.2 (Robinson et al., 2010).

Fig 1

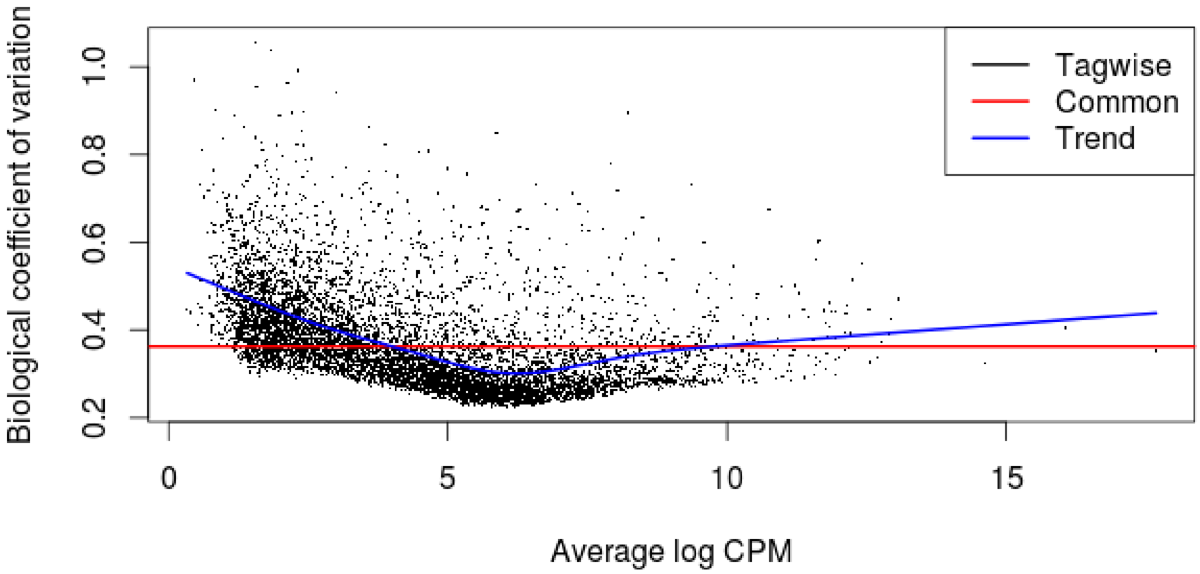
Biological coefficient of variation
0.2
0.4
0.6
0.8
1.0
0
5
10
15
Average log CPM
Tagwise
Common
Trend

Fig 2

A

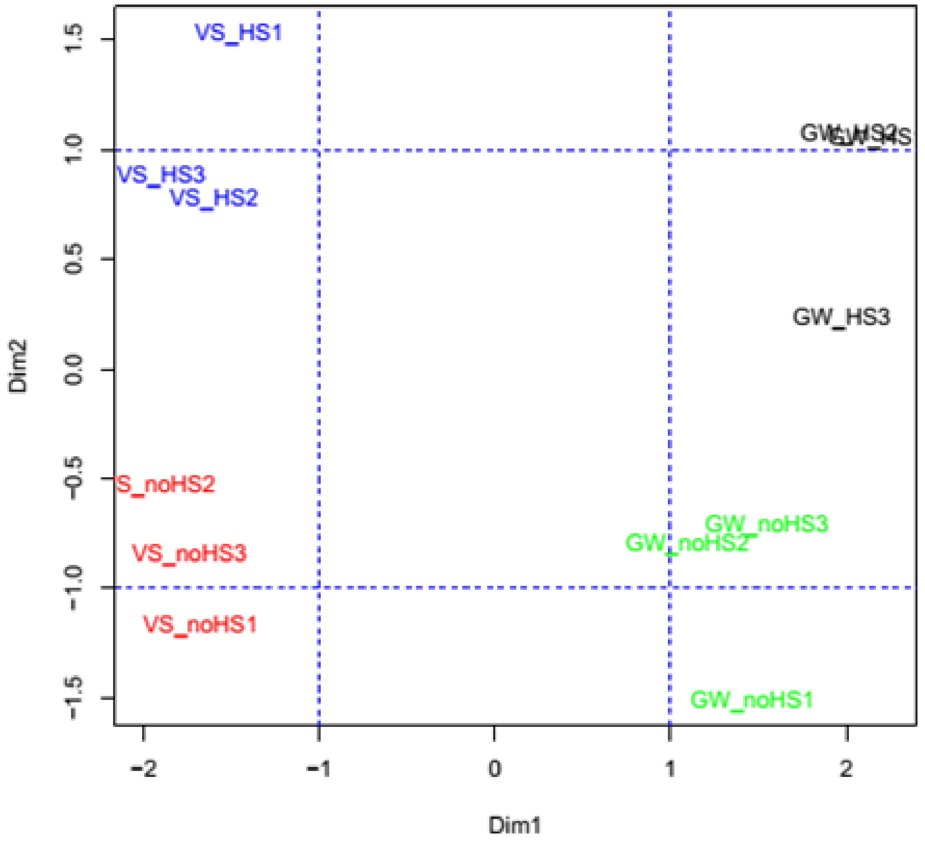
VS_HS1
VS_HS3
VS_HS2
GW_HS3
S_noHS2
VS_noHS3
VS_noHS1
GW_noHS3
GW_noHS2
GW_noHS1
1.5
1.0
0.5
0.0
−0.5
−1.0
−1.5
Dim2
−2
−1
0
1
2
Dim1

B

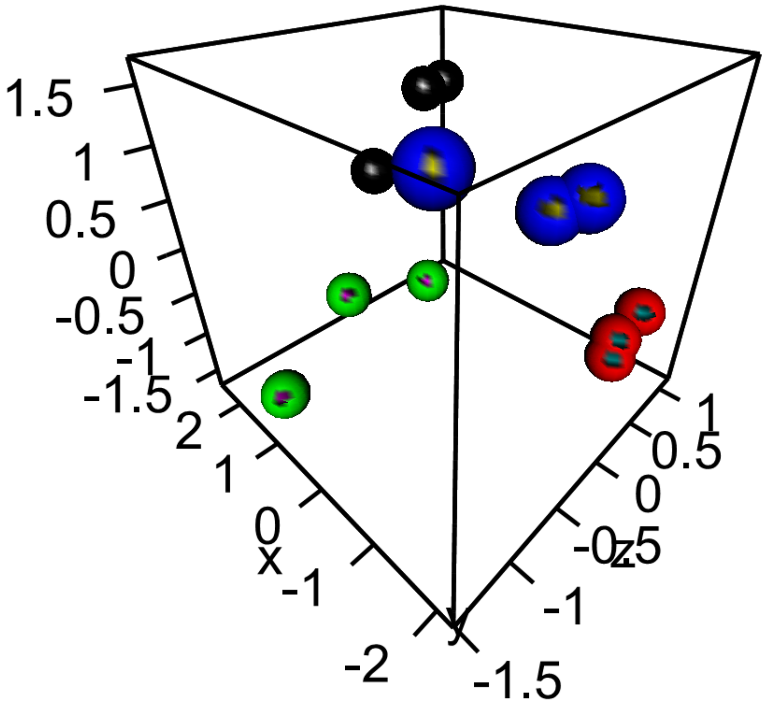
1.5
1
0.5
0
-0.5
-1
-1.5
2
1
0
x
-1
-2
y
1
0.5
0
-0.5
z
-1
-1.5

Fig 3

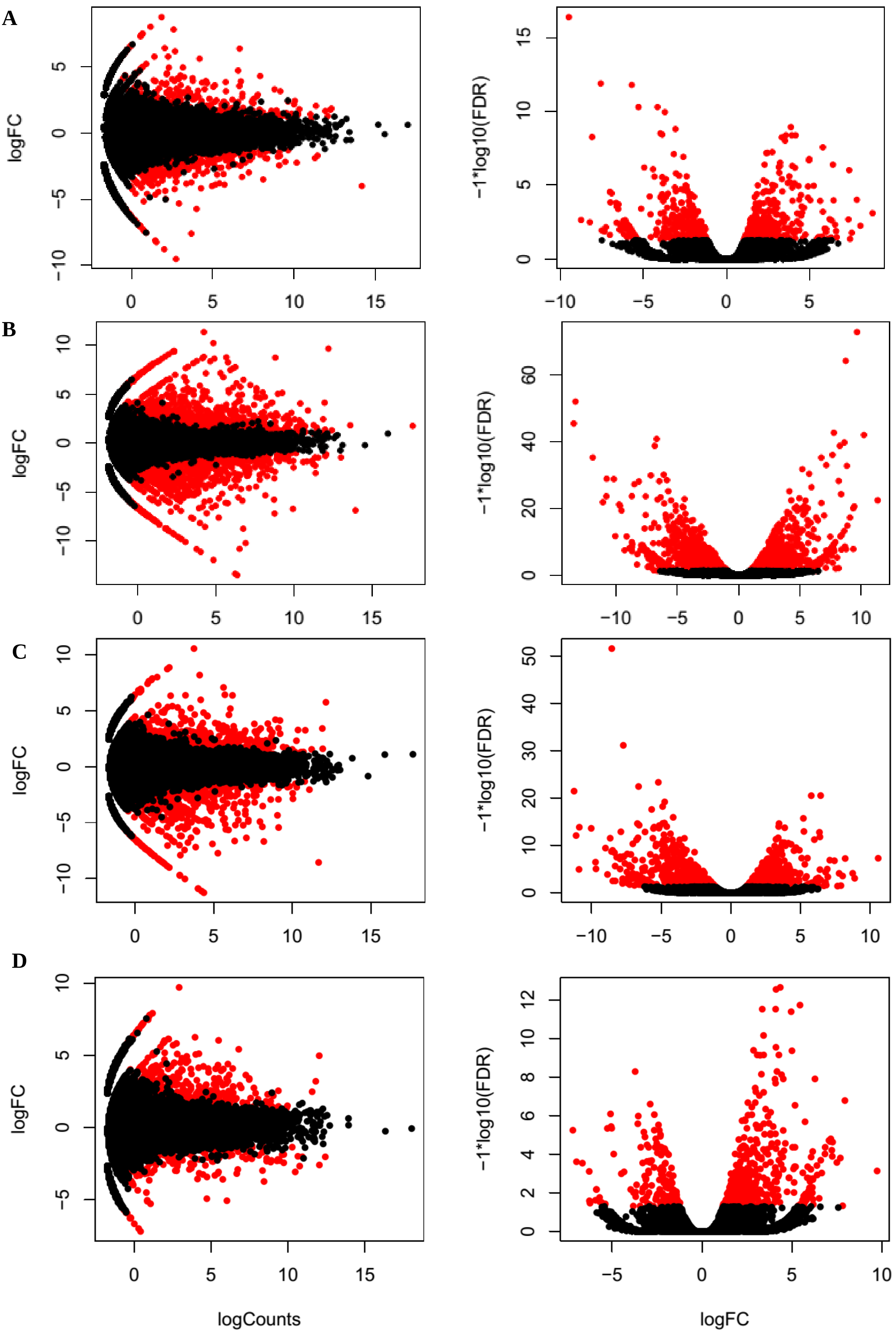

References

Adler, D., Nenadi, O., & Zucchini, W. (n.d.). RGL : A R-library for 3D visualization with OpenGL The RGL – package, 1–11.

Robinson, M., McCarthy, D., Chen, Y., & Smyth, G. (2010). edgeR: differential expression analysis of digital gene expression data, (September 2008). Retrieved from ftp://ftp2.uib.no/pub/bioconductor/2.6/bioc/vignettes/edgeR/inst/doc/edgeR.pdf
